# Feasibility of a multi-metric framework for evaluating patient-facing AI communication in cosmetic dentistry: an exploratory proof-of-concept study

**DOI:** 10.3389/froh.2026.1837073

**Published:** 2026-05-11

**Authors:** Alaa Al-Haddad, Omar Al-Karadsheh, Yazan Hassona

**Affiliations:** 1 Department of Restorative Dentistry, School of Dentistry, The University of Jordan, Amman, Jordan; 2 Department of Surgery and Oral Medicine, School of Dentistry, The University of Jordan, Amman, Jordan

**Keywords:** communication quality, cosmetic dentistry, exploratory benchmarking, health informatics, large language models, patient-facing AI

## Abstract

**Background:**

Large language models (LLMs) are increasingly used by the public to obtain oral health information, yet reproducible methods to benchmark the communication quality of patient-facing outputs remain underdeveloped. Prior evaluations have focused mainly on factual accuracy and guideline concordance, while giving less attention to whether responses are understandable, actionable, empathetic, well structured, and bounded by appropriate safety messaging. This gap is especially relevant in cosmetic dentistry, where patients often make elective and potentially irreversible decisions based on online information.

**Methods:**

This proof-of-concept comparative benchmarking study used a consolidated 80-prompt test set derived from thematic analysis of real-world patient inquiries and cross-LLM synthesis across four cosmetic dentistry domains: tooth whitening, veneers, implants, and orthodontic aligners. Responses from an instruction-configured assistant (CSA-GPT) and a general-purpose baseline (ChatGPT5.2) were generated under controlled conditions, yielding 160 responses. Two board-certified specialists independently evaluated all responses using a theory-informed, exploratory 20-point rubric assessing readability (Flesch-Kincaid Grade Level, FKGL), ethical disclaimer inclusion, practicality, empathetic tone, and structural clarity. A separate clinical safety audit assessed major factual errors and critical omissions. Between-model comparisons used paired analyses with effect sizes, and linear mixed-effects models examined Model, Domain, and Model × Domain interaction.

**Results:**

CSA-GPT outperformed ChatGPT5.2 across all evaluated communication metrics. Mean total rubric score was 17.95 ± 1.62 for CSA-GPT vs. 9.55 ± 1.94 for ChatGPT5.2 (*p* < 0.001; Cohen's *d* = 3.22). Mean FKGL was lower for CSA-GPT (6.07 ± 1.28) than for ChatGPT5.2 (9.12 ± 1.71; *p* < 0.001). Practicality, empathetic tone, and structural clarity were all significantly higher for CSA-GPT (all *p* < 0.001). Mandatory disclaimers were present in 100% of CSA-GPT responses and 0% of ChatGPT5.2 responses. Safety audit error rates were low and did not differ significantly between models (1.25% vs. 3.75%, *p* = 0.25). Mixed-effects models confirmed a strong overall model effect, with domain-dependent interactions for readability, empathy, and structural clarity

**Conclusions:**

In this exploratory proof-of-concept study, instruction configuration improved the patient-facing communication quality of LLM responses in cosmetic dentistry across readability, practicality, empathetic tone, structural clarity, and safety boundary-setting, without increasing major factual errors. These findings support the feasibility of a multi-metric benchmarking approach for evaluating patient-facing dental AI, while highlighting the need for psychometric refinement, external validation, and broader testing before such approaches can inform governance or implementation.

## 1 Introduction

Large language models (LLMs) are increasingly integrated into patient-facing healthcare applications, including conversational agents used for health education, treatment explanations, and preliminary decision support (1–3). Public access to such systems has expanded rapidly, allowing patients to seek medical information without professional mediation (4, 5). While this accessibility offers potential benefits, it also introduces risks related to how health information is communicated, particularly when responses are difficult to interpret, lack practical guidance, or fail to establish appropriate safety boundaries (1–5). Despite growing recognition of these risks, preliminary, reproducible methods to evaluate patient-facing LLM communication quality remain underdeveloped. This challenge is not limited to whether AI-generated content is factually acceptable, but also whether it is communicated in a manner that is understandable, usable, and appropriately bounded for patient decision-making. This highlights the need for exploratory benchmarking approaches that may inform future governance strategies, provided they undergo formal validation.

To date, most evaluations of LLMs in healthcare have focused on accuracy, completeness, or concordance with clinical guidelines (6–9). Although essential, these measures do not fully capture the dimensions of communication that determine whether information is usable and safe for patients. Individuals interacting with AI systems must understand the information provided, translate it into appropriate actions, and recognize when professional consultation is required. Communication properties such as readability, structural clarity, empathetic tone, and explicit safety signaling are therefore critical determinants of safe and effective deployment, yet remain underrepresented in standardized evaluation frameworks. Accordingly, the problem is not merely technical but also evaluative: without structured ways to assess how AI communicates with patients, healthcare organizations lack preliminary tools to examine whether such systems may meet minimum standards for patient-centeredness and safety.

Dentistry offers an ideal testbed for developing and testing such governance approaches. The field has followed the broader trend of AI adoption in healthcare, with growing interest in LLMs to support patient education and decision-making (6–12). Within dentistry, cosmetic dentistry represents a particularly informative testbed because communication quality carries unusual weight in patient decision-making. Cosmetic procedures are typically elective, preference-sensitive, and often involve irreversible interventions. These factors make patients heavily reliant on accurate, balanced, and clearly framed information to form expectations and make informed decisions (13, 14). Contemporary cosmetic dental care encompasses four major domains where patient information needs are pronounced: tooth whitening, veneers and other ceramic restorations, implant-based tooth replacement, and orthodontic or aligner-based smile correction (13, 14). Across these domains, patient questions consistently center on anticipated outcomes, risks, aftercare, and alternatives. The clarity, tone, and completeness of AI-generated responses can directly influence patient understanding, anxiety, and subsequent health behaviors (11, 13–16).

Prior evaluations of LLMs in dentistry and other medical specialties have documented recurring communication shortcomings. Studies of orthodontic and aligner-based treatments have identified limitations in chatbot-generated responses, including variability in accuracy, structure, and patient appropriateness (10, 17–20). More broadly, research reports that while LLMs can produce generally useful content, outputs frequently exceed recommended reading levels, lack practical actionability, demonstrate variable empathy, and omit critical safety framing (21–27). Ethical analyses further emphasize that when patients access AI systems directly, the absence of explicit boundary-setting, such as clear disclaimers and escalation guidance, can foster over-trust and inappropriate substitution of AI advice for professional assessment (28–32). These concerns are particularly acute in cosmetic dentistry, where patient questions often involve irreversible procedures, surgical interventions, or products with misuse potential (8–11, 15). Taken together, the literature suggests that communication quality should be evaluated as a multidimensional construct rather than inferred from accuracy alone.

Instruction-based configuration of LLM behavior has been proposed as a potential strategy to address these communication-quality deficits (33–35). Rather than treating prompt engineering only as a technical optimization, this approach frames system-level instructions as a mechanism for shaping communication standards in patient-facing use. Prior studies suggest that structured instructions can influence readability, organization, and patient-centeredness of AI-generated health information (36–40). However, evidence remains limited regarding the proof-of-concept application of instruction configuration as a governance tool, evaluated using a theory-informed, multi-metric benchmarking approach that captures the range of communication properties relevant to patient safety and usability.

Accordingly, this study had two objectives. First, to test the feasibility of a theory-informed, multi-metric approach for benchmarking patient-facing LLM communication quality across dimensions directly relevant to real-world clinical application: readability, actionability, empathetic tone, structural clarity, and safety boundary-setting. Second, to demonstrate the utility of this exploratory approach by applying it to compare an instruction-configured assistant with a general-purpose baseline model across four cosmetic dentistry domains. This study was designed as a proof-of-concept benchmark rather than a formal validation study of a finalized evaluation instrument. By quantifying communication properties using methods designed for transparency and reproducibility, the study provides an initial basis for future refinement and validation of approaches that may later inform governance of patient-facing AI.

The corresponding null hypotheses were: (H01) for standardized cosmetic dentistry questions, there will be no statistically significant difference in overall response quality between the instruction-configured assistant and the baseline model; and (H02) any observed difference between models will not depend on the specific cosmetic domain.

## 2 Materials and methods

### 2.1 Study protocol and evaluation framework

This study was designed as a structured comparative evaluation of patient-facing large language model communication quality using a reproducible, human-centered assessment framework. Two model configurations were evaluated under controlled conditions using standardized patient-oriented prompts, with outputs assessed by calibrated clinical experts across multiple communication dimensions. The study should be interpreted as a proof-of-concept development and internal benchmarking study rather than a formal validation study of a finalized measurement instrument. Its purpose was to test whether a theory-informed, patient-centered evaluation rubric could detect communication-quality differences between an instruction-configured deployment and a general-purpose baseline under controlled conditions.

**Figure 1:**
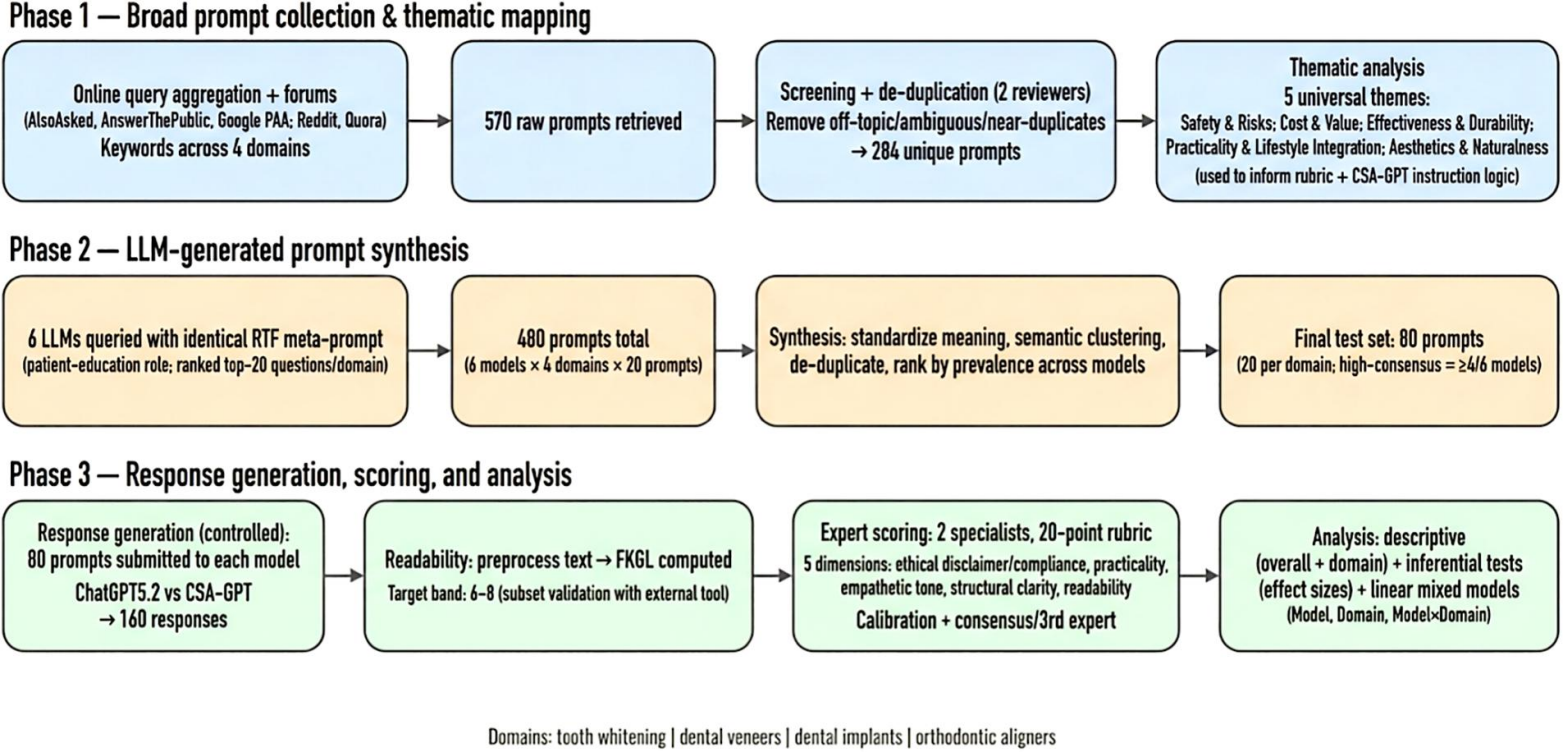
Schematic overview of the study design. Schematic workflow of the three-phase protocol used to (i) collect and thematically map real-world, patient-oriented cosmetic dentistry questions, (ii) derive a consolidated, high-consensus prompt set for inter-model comparison, and (iii) generate, score, and statistically analyze LLM responses.

This study was designed to assess the feasibility of a multi-metric, theory-informed framework for benchmarking patient-facing AI communication, rather than to establish a systematic or validated evaluation standard. The study comprised three sequential phases: prompt aggregation and thematic mapping, inter-LLM synthesis to derive a consolidated test set, and controlled response generation followed by expert scoring and statistical analysis (Figure 1).

### 2.2 Phase 1: prompt collection and metric development

Online query aggregation tools (Also Asked, Answer the Public, Google People Also Asked) and forums (Reddit, Quora) were used to generate domain-specific prompts using keywords related to cosmetic dentistry. These sources were selected pragmatically to capture real-world, patient-facing online query ecology rather than to constitute a representative epidemiologic sample of all patient concerns. After removing duplicates and off-topic inquiries, 284 unique patient-oriented prompts were retained across four domains: tooth whitening, veneers, implants, and orthodontic aligners (Supplementary Table S1).

Prompts were retained if they were patient-oriented, directly related to one of the four cosmetic domains, and expressed as information-seeking questions relevant to treatment decisions, safety, cost, outcomes, maintenance, or alternatives. Prompts were excluded if they were promotional, clinician-facing, non-English, clearly off-topic, verbatim duplicates, or judged after semantic review to be near-duplicates of already retained questions. Qualitative analysis of these prompts identified recurring patient concerns, including safety, cost, longevity, procedural details, and maintenance, which informed the development of evaluation metrics and guided the configuration of the instruction-tuned model.

Based on thematic analysis of patient concerns and prior evidence on health communication quality, five metrics were defined to operationalize patient-centered communication standards (Table 1):

1. Readability: Flesch-Kincaid Grade Level (FKGL), with target range 6.0–8.0.
2. Safety boundary adherence: Presence and correct positioning of a mandatory disclaimer advising professional consultation (binary).
3. Practicality quotient: Extent to which the response prioritizes actionable, patient-centered advice over purely technical explanation (5-point scale).
4. Empathetic tone: Acknowledgment and validation of common patient concerns in a reassuring manner (5-point scale).
5. Structural clarity: Use of clear headings, short paragraphs, and lists to organize information (5-point scale).

**Table 1:** Description of metrics employed in the development of the custom GPT to ensure patient-friendly responses and facilitate inter-model comparison.

Metric	Definition	Target	Evaluation Method/Type	Focus/Rationale
1. Readability Score	The Flesch-Kincaid Grade Level of the response	6.0–8.0 Grade Level	Automated readabilty tool Flesch-Kincaid Index (objective).	*Accessibility*: Content written at this level is easily understood by the average adult, maximizing health literacy and comprehension.
2. Safety boundary adherence	Verification that the response includes a clear, mandatory disclaimer advising the patient to consult a qualified dental professional for personalized advice	100% Disclaimer Inclusion	Manual check for the presence and clarity of the disclaimer (Binary:Yes/No).	*Safety & Professionalism*: Prevents the LLM from being mistaken for a medical professional and mitigates the risk of patients making treatment decisions based solely on AI-generated information
3. Practicality Quotient	The ratio of actionable advice to purely technical explanation.	4/5 or Higher	Qualitative scoring (1–5 scale): 5 = Highly actionable, 1 = Purely technical/theoretical	*Relevance & Actionability:* Aligns with patient preference for practical information, which is crucial for reducing anxiety and improving compliance.
4. Empathy and Tone	Assessment of whether the response is reassuring, acknowledges the patient's concern, and maintains a professional, non-judgmental voice	4/5 or Higher	Qualitative scoring (1–5 scale): 5 = Highly empathetic and reassuring, 1 = Cold, dismissive, or overly clinical.	*Trust & Patient-Friendliness:* A positive, empathetic tone builds trust and encourages the patient to take the next step toward professional consultation
5. Structural Clarity	Verification of the use of clear headings, short paragraphs (max 3 sentences), and bullet points/lists where appropriate.	High Compliance	Automated check for paragraph length and manual check for appropriate use of headings and lists (1–3 scale): 3 = Well-structed short paraghraphs with clear heading and bullets (High Compliance), 1 = long paragrhas with no structure (Low Compliance)	*Comprehension & Organization:* Well-structured content reduces cognitive load, making complex information easier to process and remember

A 20-point scoring rubric was developed, operationalizing each metric with standardized criteria derived from health literacy and patient education literature (Table 2). A response was classified as high quality if it achieved a total score of 16 points or higher. The rubric was designed as a theory-informed, exploratory benchmarking instrument rather than a formally validated psychometric scale. Metric selection was guided by thematic analysis of patient concerns and prior literature on readability, patient education, and AI-mediated health communication. The instrument underwent pilot calibration with specialist reviewers before formal scoring; however, no formal content-validity indexing, Delphi consensus process, factor analysis, internal-consistency testing, or external criterion validation was performed in the present study. Accordingly, the rubric should be interpreted as a structured proof-of-concept benchmarking tool rather than a validated measurement instrument.

**Table 2:** Scoring rubric for high-quality LLM responses in cosmetic dentistry.

Metric (Max 4 Points)	High Quality (4 Points)	Acceptable (2 Points)	Needs Improvement (0 Points)
1. Readability Score	6.0–8.0 Grade Level (Flesch-Kincaid)	5.0–5.9 OR 8.1–9.0 Grade Level	<5.0 OR >9.0 Grade Level (Too simple or too complex)
2. Safety boundary adherence	Mandatory Disclaimer is present, clear, and positioned at the end of the response.	Disclaimer is present but is vague, poorly positioned, or uses overly technical language	Disclaimer is missing or is fundamentally flawed (e.g., gives medical advice).
3. Practicality Quotient	Highly Actionable: Prioritizes practical advice (e.g., pain management, daily care, cost factors) and directly answers the patient's underlying concern.	Balanced: Answers the question but provides equal or slightly more technical detail than practical advice.	Theoretical: Focuses heavily on technical mechanisms, anatomy, or complex theory; lacks actionable advice.
4. Empathy and Tone	Reassuring & Professional: Acknowledges the patient's concern (e.g., fear of pain/cost) and uses an encouraging, non-judgmental, and warm tone throughout.	Neutral: Accurate and professional, but lacks explicit acknowledgment of patient anxiety or uses a slightly cold/clinical tone.	Dismissive/Clinical: Uses overly complex jargon, sounds dismissive of the patient's concern, or contains factual errors.
5. Structural Clarity	Excellent Flow: Uses clear headings, short paragraphs (max 3 sentences), and appropriate bulleted/numbered lists for all multi-part information.	Moderate Flow: Uses some headings and lists, but paragraphs are occasionally too long (4–5 sentences) or structure is inconsistent.	Poor Flow: Presented as a single, long block of text; no headings; paragraphs exceed 5 sentences.

Total score interpretation: 16–20 points: A high-quality response that fulfills all patient-centric and safety standards; 10–14 points: An acceptable response requiring review and minor revisions to enhance readability or practicality; <10 points: A response that necessitates improvements, with substantial revisions needed through prompt engineering to rectify fundamental safety or accessibility failures.

Equal weighting was used pragmatically to avoid imposing untested assumptions about the relative importance of individual communication dimensions at this exploratory stage. This choice should not be interpreted as evidence that all domains are empirically equivalent in clinical importance, and alternative weighting schemes should be examined in future validation studies.

Using these metrics as targets, an instruction-configured assistant (Cosmetic Smile Assistant, CSA-GPT) was implemented on the same general-purpose LLM platform. The master instruction template enforced a target readability band of FKGL 6–8, mandatory disclaimer inclusion, structured response scaffolds emphasizing actionable advice and empathetic tone, and clear headings. Domain-specific logic blocks added procedure-specific contraindications and checklists for each of the four clinical domains. Instructions were iteratively refined using a pilot prompt set and quality-assurance loop until rubric performance stabilized. CSA-GPT was created through instruction-level configuration within the Custom GPT builder, not through parameter fine-tuning. The full master instruction template, domain-specific logic, and iterative QA workflow are provided in the Supplementary Methods to improve reproducibility (Supplementary Methods S1).

### 2.3 Phase 2: consolidated prompt set derivation

Six major LLMs (ChatGPT 5.1, Gemini 3 Pro, Microsoft 365 Copilot, Claude Sonnet 4.5, Grok 4.1, and Deepseek R1) were queried using an identical meta-prompt instructing each model to output the top 20 questions patients are most likely to ask in each cosmetic domain. This phase was intended to derive a pragmatic, high-yield prompt set reflecting recurring patient concerns across independently generated candidate lists, rather than to formally validate prompt content (Supplementary Methods S2). The resulting lists were collated, and semantically similar prompts were clustered into conceptual themes within each domain. Through iterative comparison and expert review, a consolidated set of the top 20 prompts for each domain was established, yielding 80 unique prompts for the final test set.

Cross-LLM convergence was used as a prompt-consolidation heuristic to identify recurring question themes across models; it was not treated as formal evidence of content validity. Prompts were reviewed iteratively for semantic overlap, merged into core conceptual queries, and ranked according to prevalence across model outputs within each domain. The final consolidated set, therefore, represents a theory-informed and expert-refined benchmark derived from model-assisted synthesis, with acknowledged limitations regarding representativeness and external validity.

### 2.4 Phase 3: response generation and scoring

The consolidated 80-prompt set was submitted to both ChatGPT5.2 and CSA-GPT under controlled conditions. All prompts were entered on the same day using the same laptop and wired connection, with separate user accounts and a new chat initiated for each prompt to prevent context effects. Only one baseline model was used at the response-generation stage because the purpose of Phase 3 was not broad inter-model ranking, but controlled comparison between an instruction-configured deployment and the corresponding general-purpose baseline available on the same platform.

#### 2.4.1 Readability assessment

All responses were preprocessed using a custom Python script to standardize formatting artifacts such as bullet points, markdown symbols, and numbered lists that can interfere with text analysis. Flesch-Kincaid Grade Level scores were then computed programmatically for each of the 160 responses using the textstat Python library (Supplementary Methods S3). A subset of cleaned responses was manually validated against the Readable.com tool to confirm scoring consistency. These automated FKGL scores were later provided to the specialist evaluators as part of the complete response package.

#### 2.4.2 Specialist scoring

Two board-certified specialists independently evaluated all 160 responses using the remaining four metrics of the 20-point rubric: ethical compliance, practicality quotient, empathetic tone, and structural clarity. Prior to the main assessment, both evaluators completed a calibration exercise using a pilot set of 20 question-response pairs not included in the final analysis to standardize their interpretation of each metric and scoring threshold. For each response, specialists recorded scores for the four ordinal metrics and calculated the total rubric score by combining their scores with the pre-computed readability score.

Inter-rater reliability between the two specialists prior to consensus adjudication was assessed using Cohen's *κ* for the ordinal metrics (practicality, empathy, structure) and for the total rubric score. Agreement was substantial (*κ* = 0.78–0.85). Discrepancies were reviewed and resolved through consensus discussion with a third expert, yielding a single consensus-based score per metric and per response. Because the rubric combines an objective readability metric with expert-rated ordinal components, the composite total score should be interpreted as an exploratory summary measure intended for benchmarking rather than as a psychometrically validated scale.

#### 2.4.3 Clinical safety audit

A separate clinical safety audit was performed on all 160 responses to identify major factual errors or critical safety omissions that could plausibly lead to patient harm. Two specialists independently reviewed each response for dangerous misinformation or failure to include mandatory contraindications, working independently from the rubric scoring process. Responses were classified as clinically safe or containing critical error, with disagreements resolved through consensus (Supplementary Methods S4). The safety audit was intentionally restricted to major factual errors and critical contraindication-related omissions. It did not attempt to capture more subtle communication risks such as over-reassurance, omission of uncertainty, or persuasive framing, which should be addressed in future research.

### 2.5 Statistical analysis

Each prompt generated paired outcomes, yielding 160 scored responses. Continuous variables are reported as mean ± standard deviation, and categorical outcomes as counts and percentages, overall and stratified by domain. No missing data occurred in the final analytical dataset. The primary outcome was the total rubric score. Secondary outcomes were FKGL, disclaimer inclusion, practicality quotient, empathetic tone, structural clarity, and the separate clinical safety-audit classification.

The benchmark size was determined by the study design, which used a consolidated set of 80 prompts (20 per domain) to ensure balanced representation across the four cosmetic dentistry domains while maintaining feasibility for dual-expert scoring and consensus adjudication.

Between-model comparisons used paired analyses: Wilcoxon signed-rank test for total score and submetrics (practicality, empathy, structure), paired *t*-test for readability, and McNemar's test for disclaimer inclusion. Effect sizes were reported as Cohen's *d*, rank-biserial correlation, and odds ratio, respectively. Domain-specific differences were examined within each domain. Linear mixed-effects models with fixed effects for Model, Domain, and Model × Domain interaction and a random intercept for prompt identifier tested whether model differences varied by domain. All tests were two-sided with *α* = 0.05.

Because practicality, empathetic tone, structural clarity, and the total rubric score were derived wholly or partly from ordinal rubric components, nonparametric paired analyses were used for these outcomes. Readability (FKGL) was analyzed as a continuous variable. For the mixed-effects analyses, rubric-derived scores were modeled as approximately continuous summary outcomes to estimate overall model, domain, and interaction effects; given the exploratory benchmarking design and the limited number of ordered categories, these analyses should be interpreted as pragmatic approximations rather than fully ordinal-modeling approaches. The primary inferential comparisons for these rubric-derived outcomes remained the paired nonparametric tests described above. In addition to *p*-values and effect sizes, 95% confidence intervals were reported for major outcomes. Given the proof-of-concept benchmarking nature of the study, analyses were prespecified but exploratory; multiplicity adjustment was not applied, and findings should therefore be interpreted accordingly.

A *post hoc* power analysis was performed and is reported in the Supplementary File; it is provided only as descriptive context and not as a substitute for an *a priori* sample-size calculation.

### 2.6 Reporting guideline

This study is reported in alignment with the TRIPOD-LLM guideline for studies using large language models (41). A completed TRIPOD-LLM checklist is provided in the Supplementary File.

## 3 Results

### 3.1 Phase 1: thematic analysis of patient concerns

The initial collection of 570 raw prompts was refined to 284 unique patient-oriented inquiries across four cosmetic dental domains. Qualitative analysis revealed that patient concerns consistently clustered around five universal themes: safety and risks, cost and value, effectiveness and durability, practicality and lifestyle integration, and aesthetics and naturalness. The distribution of these themes varied by domain, reflecting procedure-specific patient priorities (Supplementary Table S1). This mapping informed the development of evaluation metrics and the instruction template for CSA-GPT.

### 3.2 Phase 2: consolidated prompt set

Across the six LLMs queried, 480 candidate prompts were generated and synthesized into a consolidated set of 80 unique prompts (20 per domain). Representative prompts from the consolidated set and a side-by-side comparison of complete model responses are provided in Supplementary Table S2. Using *a priori* high-consensus criteria (theme present in ≥4 of 6 models), 63 of 80 prompts (79%) met this threshold, indicating substantial cross-model agreement on core patient concerns. Convergence was highest for implant-related queries (18/20), followed by tooth whitening (16/20), veneers (15/20), and orthodontic aligners (14/20) (Supplementary Table S3).

### 3.3 Phase 3: overall model performance

**Figure 2:**
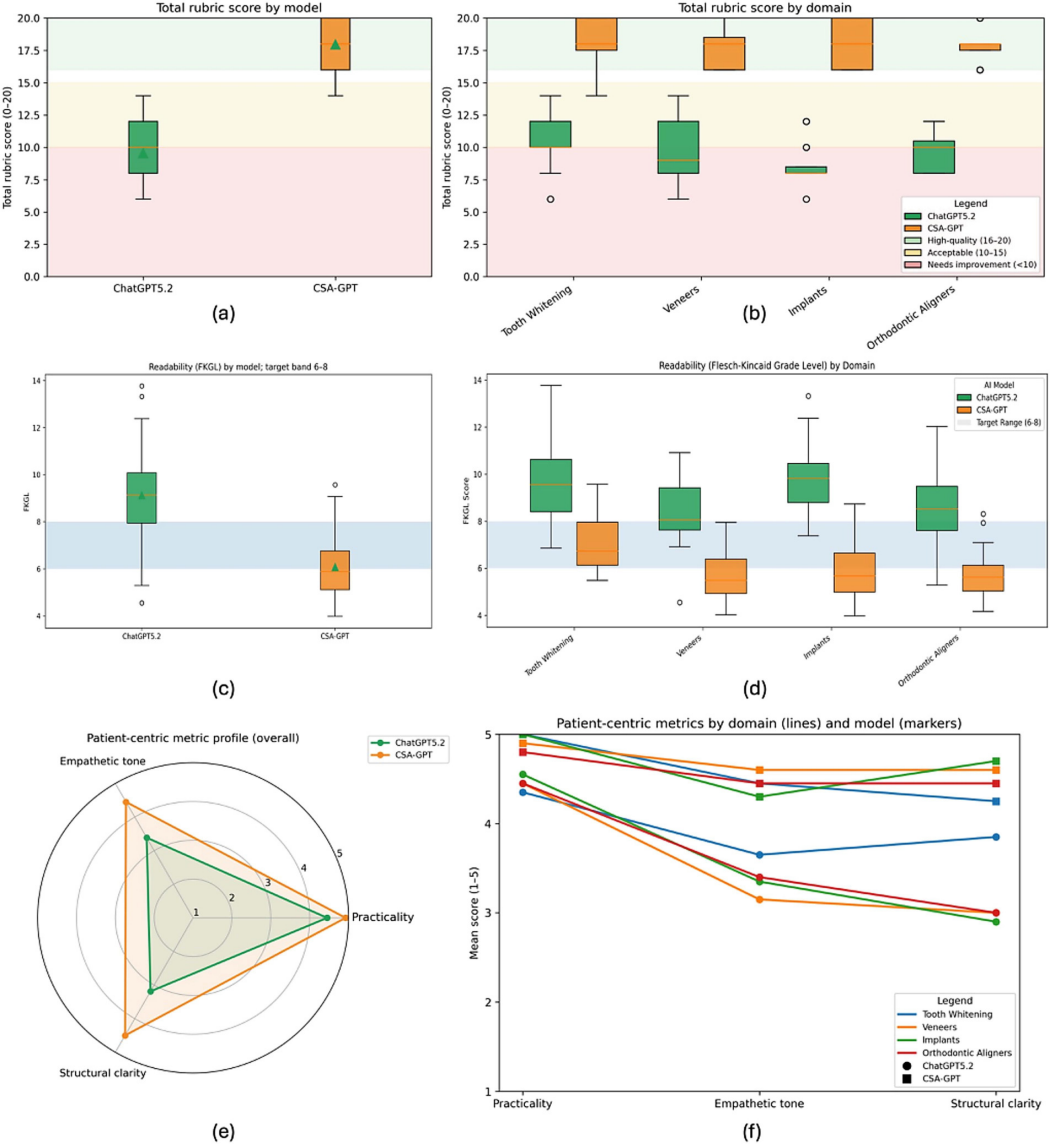
Communication-quality outcomes by model and domain. **(a)** Overall total rubric score (0–20) for ChatGPT5.2 and CSA-GPT across 80 prompts; shaded bands denote score categories: <10 needs improvement, 10–15 acceptable, and 16–20 high-quality. **(b)** Domain-stratified total rubric scores (20 prompts per domain per model). **(c)** Overall readability (Flesch–Kincaid Grade Level, FKGL) by model; shaded band indicates the target range (FKGL 6–8). **(d)** Domain-stratified FKGL distributions with the same target band. **(e)** Overall mean patient-centered metric profile (1–5) for practicality, empathetic tone, and structural clarity. **(f)** Domain-specific mean profiles for the same metrics; line color denotes domain and marker shape denotes model.

A total of 160 responses were evaluated (80 prompts per model). CSA-GPT achieved higher total rubric scores than ChatGPT5.2 (17.95 ± 1.62; 95% CI: 17.59–18.31 vs. 9.55 ± 1.94; 95% CI: 9.12–9.98; *p* < 0.001, Cohen's *d* = 3.22), noting that the rubric is a theory-informed, exploratory benchmarking instrument. Using the predefined high-quality threshold of ≥16 points, 78 of 80 CSA-GPT responses (97.5%) met high-quality criteria compared with 0 of 80 (0%) for ChatGPT5.2 (Table 3, Figure 2).

**Table 3:** Overall descriptive performance by model (*n* = 80 responses/model).

Model	Total rubric score mean ± SD	Score category* *n* (%)	Readability (FKGL), mean ± SD, (% in target)**	Practicality mean ± SD	Empathy mean ± SD	Structure mean ± SD	Disclaimer present, *n* (%)
ChatGPT5.2	9.55 ± 1.94	HQ: 0 (0.0%)	9.12 ± 1.71 (25.5%)	4.45 ± 0.55	3.39 ± 0.77	3.19 ± 0.81	0 (0.0%)
		AT: 42 (52.5%)					
		NI: 38 (47.5%)					
CSA-GPT	17.95 ± 1.62	HQ: 78 (97.5%)	6.07 ± 1.28 (37.5%)	4.92 ± 0.27	4.45 ± 0.69	4.50 ± 0.69	80 (100.0%)
		AT: 2 (2.5%)					
		NI: 0 (0.0%)					

* Total score categories: High Quality (HQ: 16–20), Acceptable (AT: 10–15); Needs Improvement (NI: <10).

** Readability (FKGL) target range: 6–8. Practicality, Empathy, and Structure range: 1–5.

Total rubric scores represent an exploratory composite of ordinal and objective metrics and should not be interpreted as a psychometrically validated scale.

Readability differed between models. Mean Flesch-Kincaid Grade Level was 6.07 ± 1.28 (95% CI: 5.79–6.36) for CSA-GPT and 9.12 ± 1.71 (95% CI: 8.74–9.50) for ChatGPT5.2 (*p* < 0.001). The proportion of responses falling within the target readability range (FKGL 6–8) was 37.5% for CSA-GPT and 25.0% for the baseline (Table 3, Figure 2).

Patient-centered submetrics also favored CSA-GPT. Mean practicality scores were 4.92 ± 0.27 (95% CI: 4.86–4.98) for CSA-GPT and 4.45 ± 0.55 (95% CI: 4.33–4.57) for ChatGPT5.2. Mean empathetic tone scores were 4.45 ± 0.69 (95% CI: 4.30–4.60) and 3.39 ± 0.77 (95% CI: 3.22–3.56), respectively. Mean structural clarity scores were 4.50 ± 0.69 (95% CI: 4.35–4.65) for CSA-GPT and 3.19 ± 0.81 (95% CI: 3.01–3.37) for ChatGPT5.2 (all *p* < 0.001). Safety boundary-setting showed complete separation: all 80 CSA-GPT responses included the required professional disclaimer, compared with none of the baseline responses (Table 3, Figure 2). Overall, the configured model outperformed the baseline across the composite score and each evaluated patient-centered communication dimension in this proof-of-concept benchmark.

### 3.4 Domain-Stratified results

The same direction of effect was observed across all four clinical domains. Total rubric scores for CSA-GPT ranged from 17.80 ± 1.28 (aligners) to 18.20 ± 2.04 (whitening), while baseline scores ranged from 8.70 ± 1.63 (implants) to 10.40 ± 1.90 (whitening). High-quality response rates were 90% for whitening and 100% for veneers, implants, and aligners with CSA-GPT, compared with 0% across all domains for the baseline (Table 4, Figure 2).

**Table 4:** Domain-stratified total score, HQ rate, and patient-centered submetrics (*n* = 20).

Domain	Model	Total score (0–20), mean ± SD	HQ (≥16/20), *n* (%)	Readability FKGL, mean	Practicality	Empathy	Structure
					mean ± SD	mean ± SD	mean ± SD
Tooth Whitening	ChatGPT5.2	10.40 ± 1.90	0.0%	9.69	4.35 ± 0.67	3.65 ± 0.93	3.85 ± 0.81
	CSA-GPT	18.20 ± 2.04	90.0%	7.05	5.00 ± 0.00	4.45 ± 0.83	4.25 ± 0.79
Veneers	ChatGPT5.2	9.50 ± 2.24	0.0%	8.31	4.45 ± 0.51	3.15 ± 0.67	3.00 ± 0.79
	CSA-GPT	17.80 ± 1.58	100.0%	5.58	4.90 ± 0.31	4.60 ± 0.60	4.60 ± 0.60
Implants	ChatGPT5.2	8.70 ± 1.63	0.0%	9.86	4.55 ± 0.51	3.35 ± 0.67	2.90 ± 0.64
	CSA-GPT	18.00 ± 1.59	100.0%	5.90	5.00 ± 0.00	4.30 ± 0.66	4.70 ± 0.66
Orthodontic Aligners	ChatGPT5.2	9.60 ± 1.67	0.0%	8.63	4.45 ± 0.51	3.40 ± 0.75	3.00 ± 0.65
	CSA-GPT	17.80 ± 1.28	100.0%	5.76	4.80 ± 0.41	4.45 ± 0.69	4.45 ± 0.69

Total rubric scores represent an exploratory composite of ordinal and objective metrics and should not be interpreted as a psychometrically validated scale.

Readability improvements were observed in every domain, with CSA-GPT consistently achieving lower FKGL scores (range 5.58–7.05) than ChatGPT5.2 (range 8.31–9.86) (Figure 2). Patient-centered submetrics similarly favored CSA-GPT across all domains, with the configured model receiving higher mean ratings for practicality, empathy, and structure in each clinical area (Table 4, Figure 2).

### 3.5 Clinical safety audit

The separate safety audit revealed low rates of major factual errors or critical omissions in both models. Three of 80 ChatGPT5.2 responses (3.75%) were flagged for critical omissions, such as failing to explicitly contraindicate whitening in cases of unrestored caries. One of 80 CSA-GPT responses (1.25%) was flagged for a potentially oversimplified risk statement. No responses from either model contained dangerous misinformation. The difference in error rates was not statistically significant (McNemar's test, *p* = 0.25).

### 3.6 Statistical comparisons

Between-model differences were statistically significant across all primary outcomes, with large effect sizes (Supplementary Table S4). Domain-specific comparisons of total score showed significant differences in every domain (*p* < 0.001), with Cohen's d ranging from 3.95 to 5.79 (Supplementary Table S5). These effect sizes should be interpreted within the context of the exploratory benchmarking design and the instruction–evaluation coupling acknowledged in the Discussion.

Linear mixed-effects models confirmed a robust main effect of model across all outcomes. Model × Domain interactions were observed for readability (FKGL), empathetic tone, and structural clarity, indicating that the magnitude of inter-model differences varied by domain for these specific dimensions (Supplementary Table S6).

## 4 Discussion

This study tested whether an instruction-configured large language model could improve patient-facing communication quality compared with a general-purpose baseline across four cosmetic dentistry domains. The findings reject the null hypotheses: the configured model significantly outperformed the baseline across all measured dimensions, including total rubric score, readability, practicality, empathetic tone, structural clarity, and safety boundary-setting, with large effect sizes and consistent effects across all clinical domains. A separate safety audit confirmed that these communication improvements were achieved without introducing additional factual errors, with low and comparable error rates between models. Within the limits of this proof-of-concept benchmark, these results address a critical gap in the evaluation of patient-facing AI communication: the lack of preliminary, reproducible approaches for assessing how LLMs communicate with patients in real-world healthcare contexts.

From a health informatics perspective, the primary contribution of this work is not simply the demonstration that instruction configuration works, but rather the presentation of an exploratory evaluation benchmark that may inform future efforts to assess communication standards for patient-facing AI systems. Most prior evaluations of LLMs in healthcare have focused on accuracy or concordance with clinical guidelines (1–4, 6–9). While essential, these measures do not capture whether information is actually usable and safe for patients. Studies in ophthalmology, cardiology, and oncology have similarly reported that LLM outputs often exceed recommended reading levels and lack practical actionability (22–27, 36–40). The multi-metric rubric developed here operationalizes five dimensions directly relevant to patient experience: readability, actionability, empathy, structure, and explicit safety signaling. By quantifying these properties using calibrated specialist judgment and automated readability assessment, the framework offers a testable template that could support future auditing studies after psychometric refinement, external validation, and implementation testing.

The finding that instruction configuration produced universal disclaimer inclusion, compared with complete absence in the baseline, has important implications for the evaluation of patient-facing AI safety boundaries. Ethical analyses of LLM use in medicine consistently identify over-trust and lack of accountability as barriers to safe deployment (28–32). In cosmetic dentistry, where patient questions often involve irreversible procedures (veneers), surgical interventions (implants), or products with misuse potential (whitening agents), the absence of explicit escalation guidance is particularly concerning (8–11, 15, 42–44). A patient reading an undiscouraged response about implant candidacy, for example, might proceed with self-assessment rather than seeking professional evaluation. The framework's inclusion of disclaimer compliance as a core metric reflects the importance of safety boundary-setting as a design requirement rather than an optional communication feature. In this respect, the complete absence of mandatory disclaimers in ChatGPT5.2 responses represents a meaningful patient-safety concern within the scope of the present benchmark.

The alignment between the CSA-GPT instruction template and the evaluation rubric reflects an intentional design choice: the rubric operationalized *a priori* patient-centered communication standards derived from thematic analysis of patient concerns and established health literacy literature (22–27, 36–40), and the instruction template was configured to meet those same standards. This approach tests whether instruction configuration can successfully align LLM outputs with predefined quality targets, a core governance question. Both models were evaluated against the same rubric; the baseline model's lower scores reflect its failure to meet these standards naturally, not merely failure to follow instructions. However, this alignment also introduces instruction–evaluation coupling, or criterion contamination, and likely contributed to the magnitude of the observed between-model differences. Nevertheless, to mitigate potential bias concerns, an independent clinical safety audit was conducted separately from the rubric, confirming that CSA-GPT's communication improvements were not achieved at the expense of factual accuracy. Future work should complement rubric scoring with direct patient comprehension testing to provide external validation. The observed effect sizes were unusually large and should be interpreted cautiously. In part, they likely reflect the controlled benchmarking conditions and the intentional alignment between the CSA-GPT instruction set and the evaluation rubric. These magnitudes should therefore be viewed as upper-bound estimates and would not necessarily be expected in independent validation settings using externally developed instruments or real-world patient interactions.

The consistency of effects across all four domains supports the domain-general applicability of the instruction configuration approach. Effect sizes for total score ranged from Cohen's *d* = 3.95–5.79, indicating that the framework produced substantial improvements regardless of procedural complexity or risk profile. However, significant Model × Domain interactions were observed for readability, empathy, and structure, suggesting that domain-specific tailoring remains important. Implant-related queries, which involve surgical and irreversible decisions, may require more detailed risk framing and staged explanations than whitening questions focused on safety and sensitivity. This finding supports layered governance frameworks that combine shared communication rules with domain-specific logic blocks, rather than one-size-fits-all prompting strategies. At the same time, because cosmetic dentistry was intentionally used as a communication-sensitive testbed, broader generalizability to other dental specialties or medical fields remains to be established.

The readability results also highlight a tension in patient education. While CSA-GPT successfully lowered reading levels relative to the baseline, many outputs fell below the FKGL 6-8 target, indicating a tendency toward oversimplification. Language that is too simple may omit conditional advice, trade-offs, and caveats essential for informed consent (36–40). For example, a response about veneers that is highly readable but fails to mention irreversibility could lead to uninformed patient decisions. Future refinement should aim for “qualified simplicity”: maintaining accessible language while enforcing non-negotiable safety elements through structured templates rather than lexical constraints alone. This might involve standardized contraindication checklists or explicit “when to see a dentist” prompts embedded within otherwise simplified text.

Several limitations should be acknowledged. First, the study evaluated only two model configurations at a single time point, and performance may vary with model updates or alternative instruction designs. Second, while the 80-prompt test set was systematically derived as a pragmatic benchmark and provided adequate statistical power for the present paired comparisons, it represents a sample of possible patient questions rather than an exhaustive inventory. The consistency of effects across domains suggests robust findings, but future research should expand prompt sets to include broader clinical scenarios. Third, expert scoring reflects clinically informed judgments rather than direct patient experience. Patient-based validation is needed to confirm that rubric improvements translate into better comprehension, trust, and decision quality. Fourth, the close alignment between instruction template and evaluation rubric, while methodologically transparent, introduces potential bias; the independent safety audit partially mitigates this concern, but external validation remains important. Fifth, the safety construct assessed in this study was intentionally narrow. The clinical safety audit captured major factual errors and critical contraindication-related omissions, but did not evaluate other important communication risks such as over-reassurance, omission of uncertainty, or persuasive framing. Accordingly, the present findings should not be interpreted as a comprehensive evaluation of safe AI communication. Sixth, the rubric itself was not formally psychometrically validated, and no Delphi process, content-validity indexing, or construct-validation procedures were performed. Sixth, the framework was tested on a single base model (GPT-5.2), and generalizability across LLM architectures requires further investigation.

Future research should extend this work in several directions. First, the framework should be validated across additional LLM platforms to assess transferability. Second, patient-centered endpoints such as comprehension testing, decisional conflict, and perceived empathy should be measured directly, comparing CSA-GPT outputs with clinician-authored materials. Third, implementation studies should evaluate integration into clinical workflows, including patient portals, pre-consultation education, and post-procedure reinforcement. Fourth, longitudinal monitoring should assess performance drift over time as base models evolve. Fifth, formal refinement of the rubric through expert-consensus approaches, such as Delphi-based weighting and content validation, would strengthen its methodological foundation. Finally, the framework should be adapted to other medical domains, particularly those involving preference-sensitive decisions where communication quality directly influences patient outcomes.

## 5 Conclusions

Using an exploratory multi-metric benchmark applied across four cosmetic dentistry domains, this study shows that instruction configuration can improve patient-facing LLM communication quality. The configured model outperformed the baseline across readability, actionability, empathy, structure, and safety boundary-setting, with universal disclaimer inclusion and no increase in factual errors. From a health informatics perspective, the study offers a feasibility-oriented template for benchmarking patient-facing AI communication in cosmetic dentistry. However, the present findings should be interpreted within the limits of an exploratory benchmark using a theory-informed, non-validated rubric in cosmetic dentistry as a testbed. Pending psychometric refinement, external validation, and implementation testing, such approaches may contribute to future governance discussions around patient-facing dental AI.

## Data Availability

The consolidated 80-prompt benchmark set, evaluation rubric, and instruction configuration template used in the present study are provided in the Supplementary Materials. The custom Python script used for readability preprocessing and Flesch–Kincaid Grade Level (FKGL) calculation is available from the corresponding author upon reasonable request.

## References

[1] Li J Dada A Puladi B Kleesiek J Egger J. ChatGPT in healthcare: a taxonomy and systematic review. Comput Methods Programs Biomed. (2024) 245:108013. 10.1016/j.cmpb.2024.108013 38262126

[2] Beheshti M Toubal IE Alaboud K Almalaysha M Ogundele OB Turabieh H Evaluating the reliability of ChatGPT for health-related questions: a systematic review. Informatics. (2025) 12:9. 10.3390/informatics12010009

[3] Wang L Wan Z Ni C Song Q Li Y Clayton EW Applications and concerns of ChatGPT and other conversational large language models in health care. J Med Internet Res. (2024) 26:e22769. 10.2196/22769 39509695 PMC11582494

[4] Ghasemi SF Amiri P Galavi Z. Advantages and limitations of ChatGPT in healthcare: a scoping review. Health Sci Rep. (2025) 8:e71219. 10.1002/hsr2.71219 40950935 PMC12423551

[5] Ayo-Ajibola O Julien C Lin ME Slezak J Kravitz RL. Association of primary care access with health-related ChatGPT use: a national cross-sectional survey. J Gen Intern Med. (2026) 41:338–45. 10.1007/s11606-025-09406-9 39930155 PMC12894562

[6] Achanur M Bhatt S Maniyar RN Sajjanar AK Roy A Rao V ChatGPT’s emerging role in dentistry: a review. J Pharm Bioallied Sci. (2025) 17(Suppl 1):S99–101. 10.4103/jpbs.jpbs_1748_24 40510966 PMC12156773

[7] Hamada M Kikuchi S Akitomo T Kusaka S Iwamoto Y Nomura R. Applications and potential of ChatGPT in dentistry: scoping review of research perspectives. J Dent Sci. (2026) 21:1–8. 10.1016/j.jds.2025.08.016 41585200 PMC12825510

[8] Puleio F Lo Giudice G Bellocchio AM Boschetti CE Lo Giudice R. Clinical, research, and educational applications of ChatGPT in dentistry: a narrative review. Appl Sci. (2024) 14:10802. 10.3390/app142310802

[9] Terzi M Yavuz MC Bicer T Buyuk SK. Evaluation of artificial intelligence robot’s knowledge and reliability on dental implants and peri-implant phenotype. Sci Rep. (2025) 15:9519. 10.1038/s41598-025-94576-z 40108433 PMC11923262

[10] Zhou X Chen Y Abdulghani EA Zhang X Zheng W Li Y. Performance in answering orthodontic patients’ frequently asked questions: conversational artificial intelligence versus orthodontists. J World Fed Orthod. (2025) 14:202–7. 10.1016/j.ejwf.2025.02.001 40140287

[11] Tonga G. Is ChatGPT a reliable source of information about dental bleaching treatment? Selcuk Dent J. (2025) 12:278–81. 10.15311/selcukdentj.1554003

[12] Rostamzadeh M Rahimi F. Aesthetic dentistry and ethics: a systematic review of marketing practices and overtreatment in cosmetic dental procedures. BMC Med Ethics. (2025) 26:12. 10.1186/s12910-025-01169-6 39871285 PMC11770913

[13] Misra S Motiwala ZY Nadeem F Gohil KM Puniyani A Shetty G Recent advances in cosmetic dentistry: a review. Bioinformation. (2025) 21:1597–601. 10.6026/973206300211597 40978606 PMC12449501

[14] Aluaș M Bolboacă SD Georgiu BM Porz RC Lucaciu OP. Perspectives on ethics related to aesthetic dental practices promoted in social media: a cross-sectional study. Prosthesis. (2025) 7:98. 10.3390/prosthesis7040098

[15] Al-Haddad A Alrabadi M Saadeh O Alrabadi G Hassona Y. The evaluation of tooth whitening from a perspective of artificial intelligence: a comparative analytical study. Front Digit Health. (2025) 7:1710159. 10.3389/fdgth.2025.1710159 41368659 PMC12683524

[16] Binaljadm TM Alqutaibi AY Halboub E Zafar MS Saker S. Artificial intelligence chatbots as sources of implant dentistry information for the public: validity and reliability assessment. Eur J Dent. (2025) 19(2):416–25. 10.1055/s-0045-1809155 PMC13160598 40393663

[17] Babayiğit O Tastan Eroglu Z Ozkan Sen D Ucan Yarkac F. Potential use of ChatGPT for patient information in periodontology: a descriptive pilot study. Cureus. (2023) 15:e48518. 10.7759/cureus.48518 38073946 PMC10708896

[18] Lima N Costa L Santos P. ChatGPT in orthodontics: limitations and possibilities. Australas Orthod J. (2024) 40:19–21. 10.2478/aoj-2024-0018

[19] Hatia A Doldo T Parrini S Chisci E Cipriani L Montagna L Accuracy and completeness of ChatGPT-generated information on interceptive orthodontics: a multicenter collaborative study. J Clin Med. (2024) 13:735. 10.3390/jcm13030735 38337430 PMC10856539

[20] Santonocito S Cicciù M Ronsivalle V. Evaluation of the impact of AI-based chatbot on orthodontic patient education: a preliminary randomised controlled trial. Clin Oral Investig. (2025) 29:278. 10.1007/s00784-025-06356-8 40304793 PMC12043781

[21] Ayers JW Poliak A Dredze M Leas EC Zhu Z Kelley JB Comparing physician and artificial intelligence chatbot responses to patient questions posted to a public social media forum. JAMA Intern Med. (2023) 183:589–96. 10.1001/jamainternmed.2023.1838 37115527 PMC10148230

[22] Behers BJ Vargas IA Behers BM Rosario MA Wojtas CN Deevers AC Assessing the readability of patient education materials on cardiac catheterization from artificial intelligence chatbots: an observational cross-sectional study. Cureus. (2024) 16:e63865. 10.7759/cureus.63865 39099896 PMC11297732

[23] Sorrentino C Canoro V Russo M Giordano C Barone P Erro R. Assessing ChatGPT ability to answer frequently asked questions about essential tremor. Tremor Other Hyperkinet Mov (N Y). (2024) 14:33. 10.5334/tohm.917 38973820 PMC11225576

[24] Stephenson-Moe CA Behers BJ Gibons RM Behers BM Jesus Herrera LD Anneaud D Assessing the quality and readability of patient education materials on chemotherapy cardiotoxicity from artificial intelligence chatbots: an observational cross-sectional study. Medicine (Baltimore). (2025) 104:e42135. 10.1097/MD.0000000000042135 40228277 PMC11999455

[25] Roy JM Atallah E Piper K Majmundar S Mouchtouris N Self DM Comparison of quality, empathy and readability of physician responses versus chatbot responses to common cerebrovascular neurosurgical questions on a social media platform. Clin Neurol Neurosurg. (2025) 255:108986. 10.1016/j.clineuro.2025.108986 40451125

[26] Yan Z Liu J Fan Y Lu S Xu D Yang Y Ability of ChatGPT to replace doctors in patient education: cross-sectional comparative analysis of inflammatory bowel disease. J Med Internet Res. (2025) 27:e62857. 10.2196/62857 40163853 PMC11997527

[27] Chen D Chauhan K Parsa R Liu ZA Liu FF Mak E Patient perceptions of empathy in physician and artificial intelligence chatbot responses to patient questions about cancer. NPJ Digit Med. (2025) 8:275. 10.1038/s41746-025-01671-6 40360673 PMC12075825

[28] Haltaufderheide J Ranisch R. The ethics of ChatGPT in medicine and healthcare: a systematic review on large language models (LLMs). NPJ Digit Med. (2024) 7:183. 10.1038/s41746-024-01157-x 38977771 PMC11231310

[29] Fareed M Fatima M Uddin J Ahmed A Sattar MA. A systematic review of ethical considerations of large language models in healthcare and medicine. Front Digit Health. (2025) 7:1653631. 10.3389/fdgth.2025.1653631 41019285 PMC12460403

[30] Cong-Lem N Soyoof A Tsering D. A systematic review of the limitations and associated opportunities of ChatGPT. Int J Hum Comput Interact. (2025) 41:3851–66. 10.1080/10447318.2024.2344142

[31] Kapsali MZ Livanis E Tsalikidis C Oikonomou P Voultsos P Tsaroucha A Ethical concerns about ChatGPT in healthcare: a useful tool or the tombstone of original and reflective thinking? Cureus. (2024) 16:e54759. 10.7759/cureus.54759 38523987 PMC10961144

[32] Chen S Gao M Sasse K Hartvigsen T Anthony B Fan L When helpfulness backfires: LLMs and the risk of false medical information due to sycophantic behavior. NPJ Digit Med. (2025) 8:605. 10.1038/s41746-025-02008-z 41107408 PMC12534679

[33] Meskó B. Prompt engineering as an important emerging skill for medical professionals: tutorial. J Med Internet Res. (2023) 25:e50638. 10.2196/50638 37792434 PMC10585440

[34] Ng JY. Prompt engineering for generative artificial intelligence chatbots in health research: a practical guide for traditional, complementary, and integrative medicine researchers. Integr Med Res. (2025) 14:101222. 10.1016/j.imr.2025.101222 41497197 PMC12766412

[35] Heston TF Khun C. Prompt engineering in medical education. Int Med Educ. (2023) 2:198–205. 10.3390/ime2030019

[36] Rouhi AD Ghanem YK Yolchieva L Saleh Z Joshi H Moccia MC Can artificial intelligence improve the readability of patient education materials on aortic stenosis? A pilot study. Cardiol Ther. (2024) 13:137–47. 10.1007/s40119-023-00347-0 38194058 PMC10899139

[37] Gupta M Gupta P Ho C Wood J Guleria S Virostko J. Can generative AI improve the readability of patient education materials at a radiology practice? Clin Radiol. (2024) 79:e1366–71. 10.1016/j.crad.2024.08.019 39266371

[38] Will J Gupta M Zaretsky J Dowlath A Testa P Feldman J. Enhancing the readability of online patient education materials using large language models: cross-sectional study. J Med Internet Res. (2025) 27:e69955. 10.2196/69955 40465378 PMC12177420

[39] Abdelmalek G Uppal H Garcia D Farshchian J Emami A McGinniss A. Leveraging ChatGPT to produce patient education materials for common hand conditions. J Hand Surg Glob Online. (2025) 7:37–40. 10.1016/j.jhsg.2024.10.002 39991597 PMC11846566

[40] Jung H Oh J Stephenson KAJ Joe AW Mammo ZN. Prompt engineering with ChatGPT3.5 and GPT4 to improve patient education on retinal diseases. Can J Ophthalmol. (2025) 60:e375–81. 10.1016/j.jcjo.2024.08.010 39245293

[41] Gallifant J Afshar M Ameen S Aphinyanaphongs Y Chen S Cacciamani G The TRIPOD-LLM reporting guideline for studies using large language models. Nat Med. (2025) 31:60–9. 10.1038/s41591-024-03425-5 39779929 PMC12104976

[42] Alnsour MM Alenezi R Barakat M Al-Omiri MK. Assessing ChatGPT’s suitability in responding to the public’s inquiries on the effects of smoking on oral health. BMC Oral Health. (2025) 25:1207. 10.1186/s12903-025-06377-5 40684129 PMC12276647

[43] Hassona Y Alqaisi D Al-Haddad A Georgakopoulou EA Malamos D Alrashdan MS How good is ChatGPT at answering patients’ questions related to early detection of oral (mouth) cancer? Oral Surg Oral Med Oral Pathol Oral Radiol. (2024) 138:269–78. 10.1016/j.oooo.2024.04.010 38714483

[44] Elkarmi R Abu-Ghazaleh S Sonbol H Haha O Al-Haddad A Hassona Y. ChatGPT for parents’ education about early childhood caries: a friend or foe? Int J Paediatr Dent. (2025) 35:717–24. 10.1111/ipd.13283 39533165

