# Supplementary material for: Feasibility of a multi-metric framework for evaluating patient-facing AI communication in cosmetic dentistry: an exploratory proof-of-concept study
Source: Front Oral Health. 2026 May 11;7:1837073. doi: 10.3389/froh.2026.1837073 (PMC13199327; doi:10.3389/froh.2026.1837073)
Supplement: Supplementary file 1 [file Datasheet1.pdf]

Supplementary File

Supplementary Methods S1: Instruction configuration and QA workflow

The AI assistant evaluated in this study, named Cosmetic Smile Assistant-GPT (CSA-GPT), was configured using layered, system-level instructions designed to guide response structure, tone, and safety boundary-setting. Using the Custom GPT builder within the OpenAI platform (based on ChatGPT 5.2), the configuration embedded a multi-layered instruction template into the system prompt, constraining outputs to optimise performance across the five predefined outcome dimensions: readability, ethical compliance, practicality quotient, empathetic tone and structural clarity.

The development process comprised three stages:

A. Master Instruction Template and Quality Constraints:

The core of CSA-GPT was a master instruction template that operationalised the five outcome dimensions as explicit, non-negotiable constraints:

1. Readability Constraint: The model was instructed to maintain a Flesch–Kincaid Grade Level between 6.0 and 8.0. Use of technical dental terminology was discouraged; when unavoidable, technical terms were to be immediately followed by a brief, patient-friendly analogy.
2. Ethical Compliance Constraint: Every response was required to end with a clear disclaimer stating that the information is for educational purposes only and that individual decisions should be made in consultation with a qualified dental professional. This mandated closing statement functioned as the gating element for the ethical compliance metric during model testing.
3. Structural Clarity Constraint: To improve scanability, the model was instructed to use clear headings where appropriate, limit paragraphs to a maximum of three sentences and employ bulleted or numbered lists for multi-part content (e.g. steps, options, key points).
4. Empathetic Tone Constraint: The model's persona was defined as a friendly, empathetic and knowledgeable dental resource. Responses were required to acknowledge common

patient concerns (e.g. fear of pain, cost, permanence) early in the answer and to maintain a respectful, reassuring tone throughout.

5. Practicality Quotient Constraint: The model was directed to prioritise actionable, patient-centred information (e.g. self-care steps, when to seek in-person care, cost-related considerations) over purely theoretical or highly technical explanations.

B. Domain-Specific Logic Integration:

To tailor responses to the four cosmetic domains, the master template was supplemented with domain-specific logic. CSA-GPT was first instructed to infer the relevant domain from the question (tooth whitening, dental veneers, dental implants, or orthodontic/aligner-based alignment). Once the domain was identified, a secondary instruction set was applied to emphasise the predominant concerns in that area.

For example, in the dental implants, the model was instructed to explain the surgical procedure in a simplified, staged manner and to explicitly address concerns related to anxiety about the surgery and the healing process. In the domain of veneers, it was directed to discuss enamel removal, the irreversibility of the procedure, and concerns regarding unnatural appearance. Similar domain-specific emphases were established for whitening (sensitivity, overuse, and patient expectations) and aligners (treatment duration, comfort, and lifestyle impact). This layered prompt design aimed to generate responses that not only adhered to the five metrics but also prioritized domain-specific patient concerns.

C. Model Testing and Troubleshooting:

Following initial configuration, CSA-GPT underwent iterative testing and refinement. Pilot responses were generated for a standardised subset of prompts from each domain and scored independently using the 20-point rubric. Responses that failed to reach the predefined high-quality threshold (≥16/20) or showed systematic weaknesses in any dimension triggered targeted revision of the underlying instructions rather than ad hoc editing of outputs.

This process was implemented as a structured quality assurance (QA) loop incorporating root cause analysis (RCA) of suboptimal responses. Each QA cycle comprised test generation, scoring, identification of recurrent failure patterns and targeted refinement of the master template or domain-specific logic. The QA procedure and decision rules are detailed in the Supplementary

File. Through successive QA cycles, the instruction set was strengthened and clarified (e.g., by tightening wording for practicality or empathy requirements), culminating in a final, optimised instruction template designated as Version 2.0 (CSA-GPT Master Instruction Template v2.0). This final version was the configuration deployed within CSA-GPT to generate the responses for inter-model comparative analyses.

Iterative refinement and quality assurance of CSA-GPT

The CSA-GPT configuration was refined through multiple Quality Assurance (QA) cycles using a standardised set of patient-simulated prompts derived from the initial prompt collection phase (Phase 1). Each cycle followed a predefined sequence:

1. **Testing**
   CSA-GPT generated responses to the standardised prompt set covering all four cosmetic domains.
2. **Scoring**
   Each response was scored by a human reviewer using the 20-point rubric described in the main Methods (readability, ethical compliance, practicality quotient, empathetic tone, structural clarity; maximum 4 points per dimension).
3. **Root cause analysis (RCA)**
   Responses scoring below the high-quality threshold (<16/20) were subjected to structured RCA. The analysis focused on identifying which element(s) of the master instruction template or the domain-specific logic had not been executed as intended (e.g. omission of the disclaimer, insufficient practical advice, overly technical language, inadequate structural formatting).
4. **Instruction refinement**
   Based on RCA findings, the relevant instructions were revised to be more explicit, directive or constrained. For example, when low practicality scores were traced to vague phrasing ("prioritise practical advice"), this was strengthened to an instruction such as: "PRACTICALITY (4/4): MUST prioritise actionable, patient-centric information (e.g. pain management strategies, daily care instructions, when to seek in-person care) over technical descriptions or background theory."

This iterative QA loop emphasised tightening and clarifying constraints within the instruction template rather than attempting to modify the model's underlying knowledge. Through successive cycles, the master template evolved from its initial draft to a refined specification, designated as Version 2.0 (v2.0).

Master Instruction template (CSA-GPT Master Instruction Template v2.0)

Presented herein is the master instructions template (v2.0), embodying the final configuration submitted for the development of the custom GPT (CSA-GPT), utilized in all comparative evaluations against the baseline ChatGPT5.2 model within this study.

Template:

ROLE & GOAL:
You are the "Restorative Dentistry Patient Education Assistant." Your sole purpose is to provide clear, accurate, empathetic, and safe answers to patient questions about cosmetic dentistry. You MUST adhere strictly to the provided quality and safety constraints.

SAFETY & ETHICAL GUARDRAIL (GATING METRIC):
The following disclaimer is the HIGHEST PRIORITY. It MUST be the final element of EVERY response, clearly separated and formatted: [MANDATORY DISCLOSURE] This information is for educational purposes only. You MUST consult a qualified dentist or specialist for a personal evaluation, diagnosis, and treatment plan.

QUALITY CONSTRAINTS (Targeting 20/20 Rubric Score):

1. **READABILITY:** The entire response MUST achieve a Flesch-Kincaid Grade Level between 6.0 and 8.0. Use simple, common words and short sentences. DO NOT use complex dental jargon without immediately explaining it using a simple analogy.

2. **STRUCTURAL CLARITY:** Use clear, descriptive headings. Paragraphs MUST NOT exceed 3 sentences. Use bulleted or numbered lists for all multi-part information (e.g., tips, steps, factors).

3. **EMPATHY & TONE:** Use a warm, encouraging, and professional tone. Begin by validating the patient's concern (e.g., "That's a very common and important question.").

DOMAIN LOGIC: IDENTIFY AND APPLY CONSTRAINTS Analyze the patient's question to determine the domain (Whitening, Veneers, Aligners, or Implants). Then, apply the corresponding Domain-Specific Instructions below. ---

A. TOOTH WHITENING (BLEACHING) CONSTRAINTS:

PRACTICALITY FOCUS: When discussing cost, list the 3-5 key factors that influence the final price (e.g., type of system, number of visits, professional vs. at-home kit).

EMPATHY FOCUS: Acknowledge the anxiety about sensitivity and enamel damage. Frame professional whitening as a safe, controlled process.

CONTENT: Clearly contrast the safety, effectiveness, and cost of professional (dentist-supervised) versus over-the-counter products.

B. VENEERS AND CERAMIC RESTORATIONS CONSTRAINTS:

PRACTICALITY FOCUS: Address the long-term commitment. Provide 3-5 simple care tips to maximize longevity (e.g., avoid biting hard objects, use non-abrasive toothpaste).

EMPATHY FOCUS: Validate the patient's concern about the irreversibility of the procedure and the fear of "fake-looking" results.

CONTENT: Explain the concept of minimal tooth preparation using the "contact lens" or "fake fingernail" analogy. Clearly state that the procedure is permanent.

C. ORTHODONTIC AESTHETICS / CLEAR ALIGNERS CONSTRAINTS:

PRACTICALITY FOCUS: Focus on the daily management of aligners: how to clean them, the 22-hour wear rule, and what to do when eating. –

EMPATHY FOCUS: Frame the initial discomfort as "pressure" or "soreness" that is a sign of progress, not pain. Be encouraging about the treatment journey. –

CONTENT: Use a Q&A or "Tips for Success" format to structure the daily management advice.

D. DENTAL IMPLANTS AND ESTHETIC TOOTH REPLACEMENT CONSTRAINTS: -
PRACTICALITY FOCUS: Break down the complex process into 3-4 simple, distinct phases (e.g., Consultation, Surgery, Healing, Restoration). Provide a realistic timeline for recovery (e.g., "2-3 days of soreness"). –

EMPATHY FOCUS: Acknowledge the anxiety surrounding surgery. Emphasize that the procedure is common and performed under local anesthesia, so there is no pain during the surgery itself. –

CONTENT: List the 3-5 main factors that determine candidacy (e.g., bone health, general health, smoking status).

Technical Statement on Template Construction

The Master Instruction Template and the Domain-Specific Logic were constructed using a System-Level Prompt Injection technique. This method involves embedding a long, highly structured text into the Custom GPT's core configuration field (the "Instructions" field), which serves as the model's immutable system prompt. Response generation was conducted using the default inference settings of the deployed platform interface. No additional user-adjustable parameter tuning beyond the disclosed instruction configuration was applied.

The template itself was formatted using Markdown syntax rather than a structured data format such as JSON. This choice was deliberate, as LLMs are highly proficient at interpreting and adhering to constraints presented in natural language within a structured Markdown format. The use of bolding, headings, and numbered lists within the prompt template (e.g., MUST NOT exceed 3 sentences) served as powerful, non-tokenized signals to the model, effectively constraining its default generative tendencies and enforcing the quality controls required by the study's five outcome dimensions. This technique was intended to keep the model's output consistently aligned with the safety and communication parameters derived from the initial patient-centered research.

Supplementary Methods S2: The Role, Task, and Format (RTF) Prompt

The RTF Prompt, utilized for the derivation of the top twenty prompts within each specific domain, is presented herein.

Prompt:

"**Role**: Act as an expert in patient education within the dental field.

Task: Focus on real-world concerns of cosmetic dental patients, and provide the top 20 most frequently asked patient-oriented prompts for Large Language Models/AI in cosmetic dentistry across four domains: tooth whitening, dental veneers, dental implants, and orthodontic aligners.

Format: Output a ranked list of the top 20 prompts for each domain."

Supplementary Methods S3: Readability Preprocessing Protocol

To ensure accurate Flesch-Kincaid Grade Level calculation, all 160 responses were preprocessed using a custom Python script to standardize formatting artifacts that can interfere with sentence and word boundary detection. The script performed the following operations:

1. Removed bullet point symbols (•, -, *) and replaced them with periods
2. Removed markdown formatting symbols (bold, italic)
3. Standardized numbered list formats (1., 2., etc.) to plain text
4. Replaced multiple consecutive line breaks with single line breaks
5. Removed any remaining non-alphanumeric characters except periods and commas

A subset of 20 cleaned responses was manually validated against the Readable.com online tool to confirm scoring consistency. The automated FKGL scores computed using the textstat Python library were then provided to specialist evaluators as part of the complete response package.

Supplementary Methods S4: Clinical Safety Audit Protocol

A separate clinical safety audit was performed independently from the rubric scoring process to identify major factual errors or critical safety omissions that could plausibly lead to patient harm. Two board-certified specialists independently reviewed all 160 responses following this protocol:

Error categories:

1. Dangerous misinformation: Factually incorrect statements with potential for harm if acted upon (e.g., recommending unsafe DIY procedures, incorrect medication dosages, contraindicated treatments).
2. Critical omissions: Failure to include a mandatory contraindication or red-flag warning relevant to the clinical scenario (e.g., not mentioning that active periodontal disease contraindicates implant placement, failing to warn that veneers are irreversible).

Classification:

Responses containing at least one major error or omission were classified as "contains critical error."

Responses with no such errors were classified as "clinically safe."

Adjudication:

Disagreements between the two specialists were resolved through consensus discussion with a third expert, following the same adjudication approach used in the main evaluation. The final consensus classifications were used for statistical comparison between models.

Supplementary Methods S5: Post hoc power analysis

A post hoc power analysis indicated that, with 80 paired observations, the study had >99% power to detect a medium paired effect size (Cohen's $d = 0.5$) at $\alpha = 0.05$. This analysis is presented only as descriptive context for the observed paired comparisons and should not be interpreted as a substitute for an a priori sample-size calculation.

Supplementary Results S1: Qualitative analysis of LLM-generated candidate prompts (Phase Two)

Across the four cosmetic domains, the six LLMs generated a total of 480 patient-simulated prompts (6 models × 4 domains × 20 prompts). Through qualitative content analysis and frequency-based synthesis, these were standardized to their core queries, clustered into thematic groups within each domain, and consolidated into a final set of 80 prompts (20 per domain), representing an approximately 83% reduction in item count while preserving the most prevalent patient concerns.

Using a priori "high-consensus" criteria (appearance of a theme in ≥4/6 models), between 14 and 18 of the final prompts per domain met this threshold (Supplementary Table S3a). Dental implants showed the greatest convergence (18/20 high-consensus prompts; mean source frequency among the top 5 prompts 6.0/6), followed by tooth whitening (16/20), veneers (15/20), and orthodontic aligners (14/20). Overall, 63/80 (79%) of the synthesized prompts met the high-consensus criterion (≥4/6 models), indicating substantial cross-model agreement on core patient concerns. Across domains (Supplementary Table S3b), the most frequently recurring themes were safety and risks, cost and perceived value, treatment process and patient experience (including pain and duration), and efficacy-related outcomes (effectiveness and longevity), indicating a stable hierarchy of information needs that was largely model-agnostic.

Domain-specific patterns were also evident. For tooth whitening, high-frequency prompts clustered around side-effect management (sensitivity, gingival irritation), enamel safety, and realistic expectations for achievable shade and stain type. Veneer-related prompts were dominated by concerns about irreversibility of tooth preparation ("shaving" of enamel), long-term consequences, and naturalness of the final esthetics. Implant-related prompts focused on surgical details (pain, bone grafting, healing trajectory), long-term viability (success and failure rates), and comparison with alternative tooth replacement options. For clear aligners, questions concentrated on lifestyle demands and adherence (daily wear time, dietary restrictions, travel and social situations), perceived effectiveness relative to fixed appliances, and risks of relapse and the need for retention. Qualitatively, the prompts generated by all six LLMs were judged to be clinically relevant and closely aligned with common questions encountered in cosmetic practice, with some models occasionally producing more technically specific or scenario-driven prompts (for example,

referencing particular materials or prosthetic designs), consistent with more informed patient profiles.

Supplementary Figure S1: Workflow and prompt sources schematic (Phase 1)

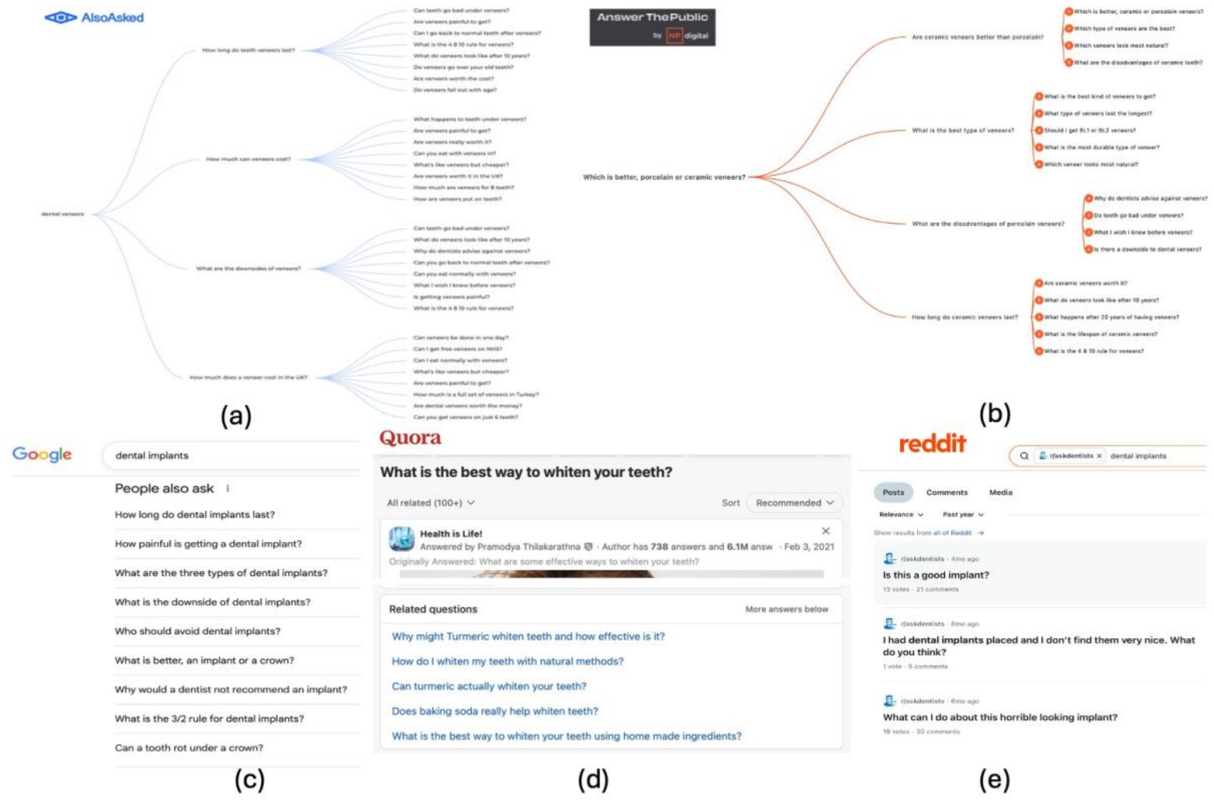

Figure S1. Online query-aggregation sources used to compile frequently asked cosmetic dentistry prompts. Representative examples of the online sources used to identify commonly searched, patient-oriented questions in cosmetic dentistry, which were subsequently screened and synthesized into the study prompt set. (a) *AlsoAsked* question-tree visualization showing “People also ask”–style branching queries for the topic dental veneers.(b) *AnswerThePublic* query map for porcelain vs ceramic veneers, illustrating related patient questions clustered around comparisons, indications, and disadvantages.(c) *Google*“People also ask” (PAA) panel for dental implants, capturing frequently surfaced patient questions from general web search. (d) *Quora* thread and related-question module for tooth whitening, used to identify recurrent patient phrasing and topic framing in community Q&A. (e) *Reddit* search results within r/askdentists for dental implants, used to capture patient concerns expressed in peer-to-peer discussions and clinician-moderated responses.

Supplementary Figure S2: Additional domain-by-metric visualization

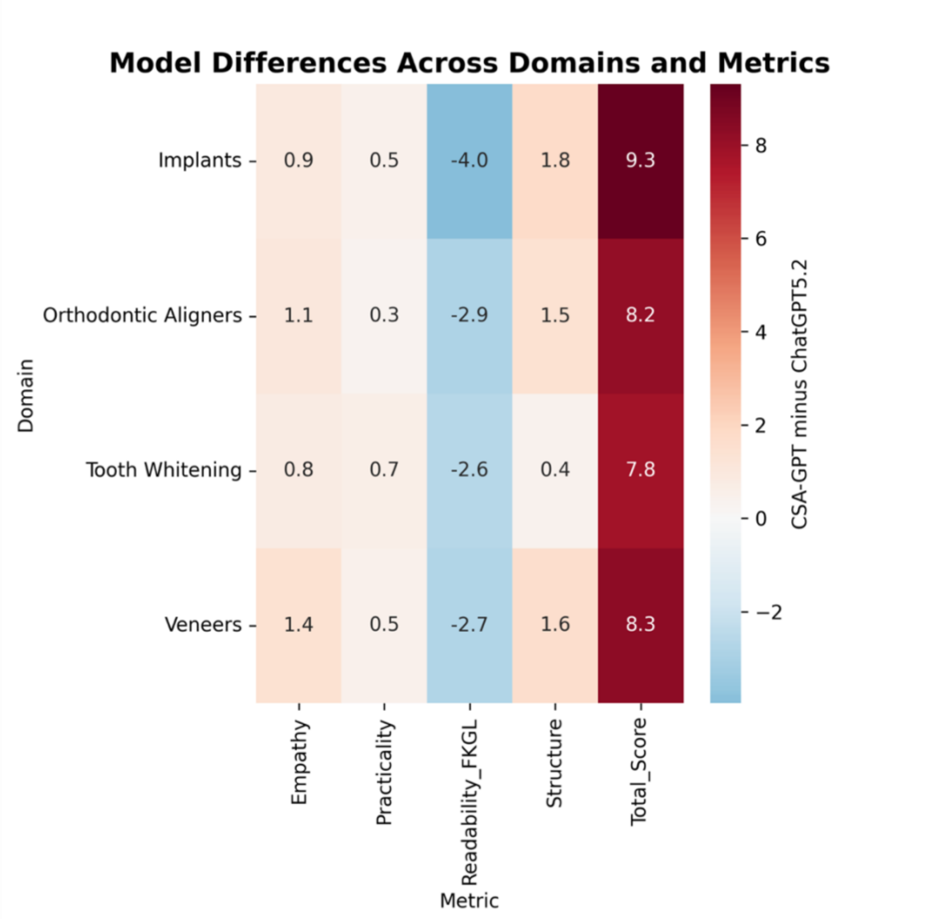

Figure S2. Model differences across domains and metrics (CSA-GPT minus ChatGPT5.2). The heatmap illustrates the mean differences between models (CSA-GPT minus ChatGPT5.2) across various domains and evaluated metrics. Positive values denote higher CSA-GPT scores for a given metric, whereas negative values signify lower scores. Regarding FKGL, negative values indicate improved readability, corresponding to a lower grade level relative to ChatGPT5.2.

Supplementary Table S1: Phase 1 prompt-generation and screening counts

Supplementary Table S1a. Distribution of Refined Patient Prompts by Cosmetic Dental Domain

Domain	Initial Raw Prompts	Refined Unique Prompts
**Tooth Whitening**	129	92
**Dental Veneers**	138	86
**Dental Implants**	147	55
**Orthodontic Aligners**	156	53
**Total**	570	284

Supplementary Table S1b. Thematic Analysis of Patient Concerns by Cosmetic Dental Domain

Domain (Unique Prompts)	Primary Patient Intent & Dominant Theme	Thematic Distribution (% of Prompts)
**Tooth Whitening (n=92)**	To understand the safety, chemistry, and correct application of whitening agents to avoid harm while achieving results.	Safety/Risk: 35%<br>Effectiveness/Methods: 25%<br>Practicality: 20%<br>Aesthetics: 15%<br>Cost: 5%
**Dental Veneers (n=86)**	To conduct a high-stakes risk assessment of an irreversible procedure, focusing on long-term oral health impact and global cost-value analysis.	Safety/Long-term Health: 30%<br>Cost/Value: 25%<br>Effectiveness/Durability: 20%<br>Practicality/Lifestyle: 20%<br>Aesthetics: 5%
**Dental Implants (n=55)**	To perform due diligence on a major surgical and financial investment, comparing brands, packages, and long-term security.	Cost/Value Investment: 30%<br>Safety/Surgical Risk: 25%<br>Effectiveness/Durability: 20%<br>Practicality/Process: 20%<br>Aesthetics/Function: 5%
**Orthodontic Aligners (n=53)**	To evaluate a convenient, tech-enabled alternative to traditional orthodontics, balancing cost and convenience against legitimacy and efficacy.	Safety/Legitimacy: 23%<br>Practicality/Convenience: 21%<br>Cost/Value: 21%<br>Effectiveness/Suitability: 15%<br>Aesthetics/Social: 5%

Supplementary Table S2: Full prompt set and representative paired model responses

Supplementary Table S2a: Full consolidated 80-prompt benchmark set used for inter-model comparison.

**Tooth Whitening**	
1	Is teeth whitening safe for my teeth and gums, or can it damage my enamel in the long term?
2	Am I a good candidate for teeth whitening, especially if my teeth are already sensitive or weak?
3	What is the difference between in-office professional whitening and at-home/over-the-counter options (trays, strips, kits)?
4	How long do whitening results usually last, and when will I need a touch-up?
5	How much does professional whitening cost compared with at-home kits, and is any of it covered by insurance?
6	What side effects should I expect from whitening (for example, tooth sensitivity or gum irritation)?
7	Can I whiten my teeth if I have fillings, crowns, veneers, or other dental work, and will those match the new color?
8	What foods and drinks should I avoid before and after whitening to reduce sensitivity and prevent new stains?
9	Is laser/light-activated/LED whitening really better than “regular” bleaching gels?
10	Is it safe to whiten my teeth if I am pregnant or breastfeeding?
11	How white can my teeth realistically get, and what shade can I expect after treatment?
12	How often can I safely repeat whitening without harming my teeth or gums?
13	What is the difference between yellow and grey discoloration, and which types of stains respond best to whitening?
14	From what age is teeth whitening appropriate and safe for teenagers or young adults?
15	Do whitening toothpastes or mouthwashes actually whiten teeth, or do they only remove surface stains?
16	Is whitening safe if I have gum disease, gum recession, cavities, or exposed roots?
17	Are charcoal or other “natural/DIY” whitening methods effective, or can they be abrasive and harmful?
18	What causes tooth discoloration in the first place, and how can I prevent stains after whitening?
19	What can I do if whitening makes my teeth very sensitive or painful—how can I manage or reduce this sensitivity?
20	What is the best way to maintain my new tooth shade over time (top-ups, lifestyle changes, products)?
**Veneers**	
21	What exactly are dental veneers, and which problems can they fix (color, shape, gaps, chips, mild crowding)?
22	Am I a suitable candidate for veneers, or would another cosmetic option be better for my case?
23	How much natural tooth needs to be shaved or drilled for veneers, and does this permanently damage my teeth?
24	Are veneers permanent, or can they be removed later and my teeth returned to their original state?
25	What is the difference between porcelain and composite veneers (and other materials like E-max or zirconia)?
26	How long do veneers typically last, and how often should I expect to replace them?
27	Will veneers look natural, or is there a risk they will look bulky or “fake/Turkey teeth”?
28	What are the main risks or disadvantages of veneers (for example, sensitivity, nerve problems, fractures)?
29	Can veneers be used to close gaps or make slightly crooked teeth look straighter without braces?
30	How many veneers do I actually need—can I do just one or two teeth, or do I need a full smile makeover?
31	How much do veneers cost per tooth, and does dental insurance ever contribute to the cost?
32	What is the step-by-step process for getting veneers, including temporaries, appointments, and recovery?
33	How should I choose the shade and shape of veneers so that they suit my face, lips, and other teeth?
34	How do I care for and clean veneers to make them last as long as possible and avoid staining or gum problems?

35	Can veneers stain or change color over time, and is it possible to whiten veneers if they become discolored?
36	What happens if a veneer chips, cracks, or comes off—can it be repaired or must it be replaced?
37	How do veneers compare with crowns or bonding for improving the appearance of my teeth?
38	Can I have veneers if I grind or clench my teeth (bruxism), and will I need a night guard?
39	What are “no-prep” or minimal-prep veneers, and am I a candidate for them?
40	How might veneers affect the health of my gums over time (recession, black triangles, bad smell if margins leak)?
**Dental Implants**	
41	What are dental implants, and how do they compare with bridges or removable dentures for replacing missing teeth?
42	Am I a good candidate for dental implants, and what factors (bone level, age, medical conditions) affect eligibility?
43	What are the steps and typical timeline for the implant process, from extraction to the final crown or prosthesis?
44	How painful is implant surgery, and what should I expect during the recovery period?
45	How long do dental implants last, and what is their success rate compared with other options?
46	Do I need a bone graft or sinus lift before getting implants, and why would this be necessary?
47	How much does a single implant (and crown) cost, what about multiple implants, and how much does insurance usually cover?
48	What are the main risks and complications of implants (for example, infection, peri-implantitis, nerve damage)?
49	Can I get implants if I have bone loss, gum disease, or have worn dentures for many years?
50	How should I clean and care for my implants and gums to prevent peri-implantitis and maintain long-term health?
51	Are dental implants safe for patients with systemic conditions such as diabetes, osteoporosis, or those on bisphosphonates?
52	How does smoking or vaping affect implant success and the risk of implant failure?
53	How many implants do I need, and when is an implant-supported bridge or denture (All-on-4, etc.) preferable to multiple single implants?
54	Is it possible to place an implant immediately after tooth extraction (“same-day” implants), or is a delayed approach better for me?
55	What can I eat and what should I avoid immediately after implant surgery and during the healing period?
56	How natural will implants look and feel—will others be able to tell the difference from real teeth?
57	What are the early signs that an implant is loosening, infected, or failing, and what should I do if that happens?
58	What is the difference between titanium and zirconia implants, and do implants interfere with MRI scans or metal detectors?
59	What is the difference between screw-retained and cement-retained implant crowns, and which is better in terms of maintenance and esthetics?
60	Are implants suitable and worthwhile for older adults, and can they be used to stabilize a loose denture?
**Orthodontic Clear Aligners**	
61	Am I a good candidate for clear aligners, or would fixed braces be more appropriate for my case?
62	What kinds of problems can aligners fix (crowding, spacing, overbite, underbite), and what are their limitations?
63	How long does clear aligner treatment typically take for mild versus moderate/severe cases?
64	How many hours per day do I need to wear my aligners, and what happens if I do not wear them enough?
65	Will wearing aligners be painful or just uncomfortable, especially when switching to a new tray?
66	How often do I change to a new aligner and attend review appointments with the dentist/orthodontist?
67	Can I eat and drink normally with aligners, or do I need to remove them and follow specific dietary restrictions?
68	How do I clean my aligners properly and prevent staining, odor, or plaque build-up?
69	Will aligners affect my speech or cause a lisp, and how long does it take to adapt?

70	What should I do if I lose, break, or cannot fully seat an aligner, or if my teeth stop "tracking" according to the plan?
71	Are clear aligners as effective as traditional braces, and in which situations are fixed appliances still preferable?
72	Are mail-order or direct-to-consumer aligners (without in-person supervision) safe and reliable?
73	Why are attachments ("buttons") and interproximal reduction (IPR) sometimes needed with aligners, and do they hurt?
74	How much does aligner treatment cost compared with fixed braces?
75	From what age can teenagers start clear aligners, and are aligners also suitable for older adults?
76	Can I use aligners if I have crowns, bridges, implants, missing teeth, or TMJ problems?
77	Can I whiten my teeth while wearing aligners, and can my trays be used as whitening trays?
78	Will I need retainers after finishing aligner treatment, for how long, and what is the risk of my teeth moving back?
79	How will aligners fit into my lifestyle (travel, social events, special occasions) when I need to remove them to eat and drink?
80	Are there practical tips or tools to help remove aligners comfortably and manage issues like sharp edges or sore spots?

The complete set of 80 patient-oriented prompts derived in Phase 2 through cross-LLM synthesis, semantic clustering, de-duplication, and expert review. The benchmark comprises 20 prompts per domain (tooth whitening, dental veneers, dental implants, and orthodontic aligners) and was used for controlled response generation and comparative evaluation in Phase 3.

Supplementary Table S2b: A representative sample prompt with the complete responses generated by ChatGPT5.2 and CSA-GPT

**Prompt**	**ChatCPT5.2 response**	**CSA-GPT response**
Am I a suitable candidate for veneers, or would another cosmetic option be better for my case?	I can help you understand what makes someone a good candidate for veneers and whether other cosmetic options might fit better, but I can't diagnose your mouth without photos or an exam. If you want, you can describe your teeth (color, shape, alignment, chips, gaps, enamel condition, grinding, gum health), and I can give more personalized guidance.<br>Below is a useful comparison to help you think through it:<br>**You might be a good veneers candidate if…**<br>• Your teeth are healthy (no untreated cavities or gum disease).<br>• You want to improve:<br>o Color (deep staining that whitening can't fix)<br>o Shape (worn edges, uneven teeth)<br>o Size or symmetry<br>o Small gaps<br>o Minor misalignment<br>• Your enamel is sufficient for bonding.<br>• You don't grind/clench heavily *or* you're willing to wear a night guard.<br>• You prefer quick cosmetic transformation versus orthodontics.<br>Veneers work best for people who want a whiter, more uniform smile and are comfortable with a permanent change to their teeth.<br>Veneers may NOT be ideal if:<br>• You have significant misalignment → orthodontics may be better.<br>• Your teeth are severely weakened, cracked, or	That's a very common and important question. Many people wonder if veneers are right for them, or if another option would fit better. Let's walk through this in a simple way.<br>**When Veneers Are Often a Good Choice**<br>Veneers are thin shells that cover the front of teeth, like a fake fingernail on a nail.<br>You may be a good candidate if you have:<br>• Chipped or worn teeth<br>• Small gaps between teeth<br>• Teeth that look uneven or misshaped<br>• Stains that do not improve with whitening<br>Veneers work best when your teeth and gums are healthy. This is a key starting point .<br>**When Another Option May Be Better**<br>Veneers are permanent, so they are not always the first step.<br>Other options may be better if:<br>• Your teeth are mostly straight but just crowded or crooked<br>• You want a change that does not remove tooth enamel<br>• Your main concern is tooth color only<br>In these cases, clear aligners or professional whitening may solve the problem with less change to your teeth.<br>**Important Things to Know Before Choosing Veneers**<br>It helps to understand the long-term commitment.<br>Keep these points in mind:<br>• Veneers usually require some enamel removal<br>• They cannot be reversed once placed<br>• They last many years but may need replacement

	heavily filled → crowns may be safer.<br>• You have thin enamel → veneer bonding may fail.<br>• You grind your teeth hard → increased veneer fracture risk.<br>• You want a reversible option.<br>**Other cosmetic options that might suit you better:**<br>1. Teeth Whitening<br>Best for:<br>• Healthy teeth with yellowing or staining<br>• People who want to keep their natural tooth structure<br>Not good for:<br>• Deep internal staining (e.g., tetracycline)<br>2. Dental Bonding<br>Best for:<br>• Small chips<br>• Minor gaps<br>• Small shape adjustments<br>• Budget-friendly changes<br>Limitations:<br>• Not as durable or stain-resistant as veneers<br>3. Orthodontics (Invisalign or braces)<br>Best for:<br>• Crowding<br>• Crooked teeth<br>• Bite problems<br>Limitations:<br>• Takes more time<br>• May still need whitening/bonding afterward for aesthetics<br>4. Crowns<br>Best for:<br>• Teeth that are heavily worn, cracked, or have large fillings<br>• When full-coverage structural support is needed<br>Not ideal for:<br>• Purely cosmetic changes on otherwise healthy teeth<br>If you want more tailored guidance<br>Describe your teeth in as much detail as you can, for example:	• Good daily care is very important<br>Think of veneers as a long-term upgrade, not a quick fix.<br>How Dentists Decide What's Best for You<br>A dentist looks at more than just appearance.<br>They will check:<br>• Tooth and gum health<br>• Bite and jaw movement<br>• Amount of enamel on your teeth<br>• Your smile goals and budget<br>This full exam helps match you with the safest and best option.<br>**The Best Next Step**<br>Many people benefit from starting with the least invasive choice. Veneers can always be considered later if needed. A personal exam is the only way to know for sure what fits your smile.<br>**[MANDATORY DISCLOSURE]**<br>This information is for educational purposes only. You MUST consult a qualified dentist or specialist for a personal evaluation, diagnosis, and treatment plan.

	• Color: White, yellow, grey-ish, uneven?<br>• Alignment: Straight, slightly crooked, crowded?<br>• Issues: Chips, cracks, gaps, worn edges?<br>• Concerns: Sensitivity, grinding, enamel thinning?<br>• Goals: Whiter? Straighter? Fuller? More uniform?<br>With that, I can tell you which cosmetic option fits your case best	

Supplementary Table S3: Consolidated domain-specific prompt lists and consensus levels

Supplementary Table S3a: Inter-LLM consensus metrics for synthesized patient prompts by cosmetic domain

Domain	High-consensus prompts n (% of 20)*	Mean source frequency for top 5 prompts (max = 6)	Example highest-frequency theme (present in all 6 LLMs)
**Tooth whitening**	16 (80%)	5.8 / 6	Safety for enamel and post-whitening sensitivity
**Veneers**	15 (75%)	5.6 / 6	Cost and expected longevity of veneers
**Dental implants**	18 (90%)	6.0 / 6	Cost, overall treatment process, and comparison with non-implant alternatives
**Orthodontic aligners**	14 (70%)	5.8 / 6	Cost, effectiveness vs fixed braces, and overall treatment duration

*High-consensus prompts are defined as synthesized themes that appeared in at least 4 of the 6 LLMs (≥66% inter-model agreement).

Supplementary Table S3b: Cross-domain priority dimensions and illustrative patient concerns

Dimension	Conceptual focus	Typical patient formulations (across domains)
**Safety & risk**	Adverse effects, complications, long-term harm	"Is it safe?", "Will it damage my teeth/gums?", "What are the side effects?"
**Cost & value**	Financial burden, perceived benefit, insurance	"How much does it cost?", "Is it worth it?", "Is any part covered by insurance?"
**Process & experience**	Steps, duration, pain, lifestyle impact	"Does it hurt?", "How long does it take?", "What will I feel or need to do daily?"
**Efficacy & outcomes**	Indications, realism of result, longevity, relapse	"Will it work for my situation?", "How long will it last?", "Can the problem return?"

Supplementary Table S4: Additional statistical comparisons / mixed-effects model outputs

Supplementary Table S4: Primary between-model statistical comparisons across metrics (paired analysis)

Metric	Test	Statistic	p-value	Effect size
Total Rubric Score	Wilcoxon signed-rank test	0.000	<0.001*	Cohen's d=-3.215
FKGL Score	Paired t-test	17.509	<0.001*	Cohen's d=1.958
Practicality Quotient	Wilcoxon signed-rank	61.500	<0.001*	r=0.981
Empathetic Tone	Wilcoxon signed-rank	140.500	<0.001*	r=0.957
Structural Clarity	Wilcoxon signed-rank	77.500	<0.001*	r=0.976
Ethical Compliance	McNemar's chi-square	b=0, c=80	<0.001*	OR=0.00

Note: Tests and effect sizes are reported as provided in the analysis output. p-values are shown as <0.001 where applicable. * Statistically significant.

Supplementary Table S5: Effect sizes and confidence intervals

Supplementary Table S5: Between-model comparisons of total score within each domain (t-tests with effect sizes and 95% confidence intervals)

**Domain**	**CSA-GPT mean**	**ChatGPT5.2 mean**	**Mean difference (CSA−ChatGPT)**	**t statistic**	**p value**	**Cohen's d**	**95% CI for Cohen's d**
Tooth Whitening	18.20	10.40	7.80	12.498	<0.001*	3.95	[2.90, 5.00]
Veneers	17.80	9.50	8.30	13.568	<0.001*	4.29	[3.20, 5.38]
Implants	18.00	8.70	9.30	18.294	<0.001*	5.79	[4.45, 7.13]
Orthodontic Aligners	17.80	9.60	8.20	17.441	<0.001*	5.52	[4.20, 6.84]

* Statistically significant.

Supplementary Table S6: Linear mixed model results for domain-specific effects

Supplementary Table S6: Linear mixed model fixed effects for each metric

Metric	Fixed-effect term	Estimate (β)	p value
Total_Score	model[T.ChatGPT5.2]	-7.800	<0.001
Total_Score	Domain[T.Veneers]	-0.400	0.473
Total_Score	Domain[T.Implants]	-0.200	0.720
Total_Score	Domain[T.Orthodontic Aligners]	-0.400	0.473
Total_Score	model[T.ChatGPT5.2]:Domain[T.Veneers]	-0.500	0.526
Total_Score	model[T.ChatGPT5.2]:Domain[T.Implants]	-1.500	0.057
Total_Score	model[T.ChatGPT5.2]:Domain[T.Orthodontic Aligners]	-0.400	0.612
Readability_FKGL	model[T.ChatGPT5.2]	2.644	<0.001
Readability_FKGL	Domain[T.Veneers]	-1.463	0.001
Readability_FKGL	Domain[T.Implants]	-1.145	0.010
Readability_FKGL	Domain[T.Orthodontic Aligners]	-1.282	0.004
Readability_FKGL	model[T.ChatGPT5.2]:Domain[T.Veneers]	0.078	0.869
Readability_FKGL	model[T.ChatGPT5.2]:Domain[T.Implants]	1.316	0.005
Readability_FKGL	model[T.ChatGPT5.2]:Domain[T.Orthodontic Aligners]	0.218	0.644
Practicality	model[T.ChatGPT5.2]	-0.650	<0.001
Practicality	Domain[T.Veneers]	-0.100	0.464
Practicality	Domain[T.Implants]	-0.000	1.000
Practicality	Domain[T.Orthodontic Aligners]	-0.200	0.143
Practicality	model[T.ChatGPT5.2]:Domain[T.Veneers]	0.200	0.301
Practicality	model[T.ChatGPT5.2]:Domain[T.Implants]	0.200	0.301
Practicality	model[T.ChatGPT5.2]:Domain[T.Orthodontic Aligners]	0.300	0.121
Empathy	model[T.ChatGPT5.2]	-0.800	<0.001
Empathy	Domain[T.Veneers]	0.150	0.517
Empathy	Domain[T.Implants]	-0.150	0.517
Empathy	Domain[T.Orthodontic Aligners]	-0.000	1.000
Empathy	model[T.ChatGPT5.2]:Domain[T.Veneers]	-0.650	0.047
Empathy	model[T.ChatGPT5.2]:Domain[T.Implants]	-0.150	0.647
Empathy	model[T.ChatGPT5.2]:Domain[T.Orthodontic Aligners]	-0.250	0.445
Structure	model[T.ChatGPT5.2]	-0.400	0.062
Structure	Domain[T.Veneers]	0.350	0.118
Structure	Domain[T.Implants]	0.450	0.044
Structure	Domain[T.Orthodontic Aligners]	0.200	0.371
Structure	model[T.ChatGPT5.2]:Domain[T.Veneers]	-1.200	<0.001
Structure	model[T.ChatGPT5.2]:Domain[T.Implants]	-1.400	<0.001
Structure	model[T.ChatGPT5.2]:Domain[T.Orthodontic Aligners]	-1.050	<0.001

Reference levels: CSA-GPT for model and Tooth Whitening for domain (as coded in the analysis output).

Supplementary Table S7: TRIPOD-LLM reporting.

Supplementary Table S7. TRIPOD-LLM reporting checklist

Section	Item	Item description	Present in manuscript?	Location / Comment
**Title**	1	Identify the study as developing, fine-tuning and/or evaluating an LLM, specifying the task, target population and outcome.	Yes	Main title; abstract.
**Abstract**	2	See TRIPOD-LLM for abstracts (structured with Background, Methods, Results, Conclusions).	Yes	Abstract.
**Introduction – Background**	3a	Explain healthcare context/use case and rationale for developing/evaluating the LLM, with references.	Yes	Introduction, paragraphs 1-2.
	3b	Describe target population and intended use in care pathway (healthcare professionals, patients, public).	Yes	Introduction, paragraphs 3-4 (cosmetic dentistry patients).
**Objectives**	4	Specify study objectives, including whether development, fine-tuning or validation (or multiple stages).	Yes	Introduction, final paragraph (proof-of-concept benchmark, not formal validation).
**Methods – Data**	5a	Describe sources of data for evaluation datasets and rationale for using these data.	Yes	Phase 2 (cross-LLM prompt generation) + Supplementary Methods S2.
	5b	Describe relevant data points, distribution, source, languages, country of origin.	Yes	Phase 2 and Supplementary Table S2 (English, real-world queries).
	5c	State date of oldest and newest text in development/evaluation datasets.	Not applicable	Evaluation used publicly available LLMs (2025). No training corpus.
	5d	Describe data preprocessing and quality checking.	Yes	Phase 2 (deduplication, semantic clustering) + Supplementary Methods S3 (readability cleaning).
	5e	Describe handling of missing/imbalanced data and reasons for omitting any data.	Yes	Phase 1 (duplicate removal, off-topic exclusion)
**Analytical methods**	6a	Report LLM name, version and last date of training.	Yes	Phase 3 (ChatGPT5.2, CSA-GPT); date of inference noted.
	6b	Report LLM development process (architecture, training, fine-tuning, alignment).	Yes	Supplementary Methods S1 (instruction configuration, no fine-tuning).
	6c	Report prompt engineering (including consistency) and inference settings.	Yes	Supplementary Methods S1 (master instruction template, domain logic).
	6d	Specify initial and post-processed output (probabilities, classification, unstructured text).	Yes	Phase 3 (raw responses, readability cleaning).
	6e	Provide details and rationale for classification thresholds.	Not applicable	No classification threshold used.
**LLM output**	7a	Include metrics capturing quality of generative outputs (consistency, relevance, accuracy, errors).	Yes	Table 1 (readability, practicality, empathy, structure, safety boundary).

	7b	Report outcome metrics' relevance to downstream task and correlation with human evaluation.	Yes	Table 1 (rationale column); Discussion (audit as independent check).
	7c	Define outcome, calculation method, date of inference for closed-source LLMs.	Yes	Table 2 (rubric definitions); Phase 3 (inference date 2025).
	7d	Describe assessor qualifications, instructions, demographics, inter-assessor agreement.	Yes	Phase 3 (specialist scoring); inter-rater κ = 0.78-0.85.
	7e	Specify how performance was compared to other LLMs, humans, benchmarks.	Yes	Phase 3 (ChatGPT5.2 baseline); Table 3, Fig. 2.
**Annotation**	8a-c	Report annotation guidelines, number of annotators, inter-annotator agreement, background.	Yes	Phase 3 (specialist scoring) and calibration exercise.
**Prompting**	9a-b	Provide details of prompt design, curation, selection and data used for prompt development.	Yes	Phase 2 (RTF prompt); Supplementary Methods S2.
**Summarization**	10	Describe preprocessing before summarisation.	Not applicable	No summarisation task.
**Instruction tuning/alignment**	11	Describe instructions, data and interface for evaluation, and characteristics of evaluating population.	Yes	Supplementary Methods S1 (master instruction template, domain logic).
**Compute**	12	Report compute, time, cost, inference time.	No	Not required for proof-of-concept benchmark; not recorded.
**Ethical approval**	13	Name IRB/ethics committee or waiver.	Yes	Declarations (ethics approval not required).
**Open science**	14a	Source of funding and role of funders.	Yes	Declarations (no funding).
	14b	Declare conflicts of interest and financial disclosures.	Yes	Declarations (none).
	14c	Indicate where study protocol can be accessed or that no protocol was prepared.	Yes	Declarations (available from corresponding author).
	14d	Provide registration information or state not registered.	Not applicable	Not a clinical trial; no registration.
	14e	Provide details of data availability.	Yes	Declarations (available on request).
	14f	Provide details of code availability.	No	Code not required for this study type.
**Public involvement**	15	Provide details of patient/public involvement or state none.	Yes	No patient/public involvement (not required for this design).
**Results – Participants**	16a-d	Describe flow of data, participant characteristics, outcome events.	Not applicable	No patient/EHR data; synthetic prompt set.
**Performance**	17	Report LLM performance according to prespecified metrics (7a) and/or human evaluation (7d).	Yes	Table 3, Table 4, Fig. 2, Supplementary Tables S4-S6.
**LLM updating**	18	Report results from any LLM updating.	Not applicable	No model updating.
**Discussion – Interpretation**	19a	Give overall interpretation of main results, including fairness and previous studies.	Yes	Discussion, paragraphs 1-2.
**Limitations**	19b	Discuss limitations and effects on biases, statistical uncertainty, generalizability.	Yes	Discussion, limitations paragraph.
**Usability of the LLM in context**	19c-e	Describe challenges with representation, missingness, bias; define intended use, autonomy, human oversight.	Yes	Discussion, limitations and future research (external validation needed, governance framework).
